# Preclinical Efficacy of VTX-0811: A Humanized First-in-Class PSGL-1 mAb Targeting TAMs to Suppress Tumor Growth

**DOI:** 10.3390/cancers16162778

**Published:** 2024-08-06

**Authors:** Tatiana Novobrantseva, Denise Manfra, Jessica Ritter, Maja Razlog, Brian O’Nuallain, Mohammad Zafari, Dominika Nowakowska, Sara Basinski, Ryan T. Phennicie, Phuong A. Nguyen, Michael A. Brehm, Stephen Sazinsky, Igor Feldman

**Affiliations:** 1 Verseau Therapeutics, 2000 Commonwealth Ave, Newton, MA 02466, USA; denise@verseautx.com (D.M.); jessica.ritter@meadowhawkbiolabs.com (J.R.); mrazlog@nextpointtx.com (M.R.); bonuallain@leveragen.com (B.O.); mohammad.zafari@alloytx.com (M.Z.); dominika.nowakowska@biontech.us (D.N.); sara.basinski@dragonflytx.com (S.B.); rphennicie@visterrainc.com (R.T.P.); anguyen@flaretx.com (P.A.N.); steve.sazinsky@verseautx.com (S.S.); 2 Diabetes Center of Excellence, UMass Chan Medical School, 368 Plantation Street, Worcester, MA 01605, USA; michael.brehm@umassmed.edu

**Keywords:** PSGL-1, macrophage, immunotherapy

## Abstract

**Simple Summary:**

Cancer can create a shield of suppressive innate immune cells that stop our body’s natural defenses from tumors. VTX-0811 is a new drug that targets these cells and turns them from allies of cancer to fighters against it. Preclinical tests show that it works in the lab on human cells and tumors and is safe and efficacious in animals. The results are promising and pave the way for human trials to see if VTX-0811 can become a new powerful weapon against cancer.

**Abstract:**

Omnipresent suppressive myeloid populations in the tumor microenvironment limit the efficacy of T-cell-directed immunotherapies, become more inhibitory after administration of T-cell checkpoint inhibitors, and are overall associated with worse survival of cancer patients. In early clinical trials, positive outcomes have been demonstrated for therapies aimed at repolarizing suppressive myeloid populations in the tumor microenvironment. We have previously described the key role of P-selectin glycoprotein ligand-1 (PSGL-1) in maintaining an inhibitory state of tumor-associated macrophages (TAMs), most of which express high levels of PSGL-1. Here we describe a novel, first-in-class humanized high-affinity monoclonal antibody VTX-0811 that repolarizes human macrophages from an M2-suppressive phenotype towards an M1 inflammatory phenotype, similar to siRNA-mediated knockdown of PSGL-1. VTX-0811 binds to PSGL-1 of human and cynomolgus macaque origins without inhibiting PSGL-1 interaction with P- and L-Selectins or VISTA. In multi-cellular assays and in patient-derived human tumor cultures, VTX-0811 leads to the induction of pro-inflammatory mediators. RNAseq data from VTX-0811 treated ex vivo tumor cultures and M2c macrophages show similar pathways being modulated, indicating that the mechanism of action translates from isolated macrophages to tumors. A chimeric version of VTX-0811, consisting of the parental murine antibody in a human IgG4 backbone, inhibits tumor growth in a humanized mouse model of cancer. VTX-0811 is exceptionally well tolerated in NHP toxicology assessment and is heading into clinical evaluation after successful IND clearance.

## 1. Introduction

Checkpoint inhibitors (CPIs) that target T-cell (re)-activation have dramatically advanced cancer treatment and led to durable responses in some patients. Despite these revolutionary treatments, the majority of patients or cancer types do not respond [1,2,3,4]. The exhausted state of the T cell [5] and the lack of T cells in multiple “cold” tumors that vastly outnumber “hot” ones [6] have been suggested as limiting CPI efficacy. Therefore, alternative immune cell targets are under investigation both on T cells [7,8] and on other immune cells including myeloid cells [9]. Myeloid cells, heavily represented by TAMs in the TME, constitute a major axis of immunosuppression [10] and are associated with poor prognosis in patients with cancer [11]. TAMs have multiple pro-tumorigenic activities, acting on immune cells and supporting tumor cells; they inhibit T-cell activation, proliferation, and effector responses through numerous mechanisms, provide nutritional and neovascularization support to the tumor, and promote metastatic progression [4].

**Figure 1:**
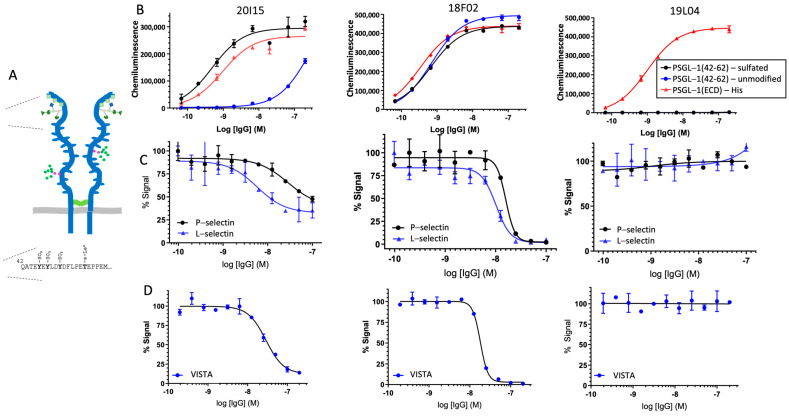
Generation of the anti-PSGL-1 antibody, 19L04. (**A**) Structure of PSGL-1: Human PSGL-1 is a homodimer linked by a disulfide-linker (green). The PSGL-1 N-terminus is responsible for binding to P-selectin, L-selectin, and VISTA and is the site of several post-translational modifications critical for ligand binding: tyrosine sulfation at residues 46, 48, and 51 and the addition of sialyl-Lewis x (inset). Between the N-terminus and the transmembrane region is the set of mucin-like domains. (**B**) Binding of anti-PSGL-1 antibodies 20I15, 18F02, and 19L04 to plate immobilized human PSGL-1-His protein, and biotinylated 21-mer peptides comprising the N-terminal amino acids of PSGL-1, residues 42–62. Sulfated peptides contain sulfated tyrosines at positions 46, 48, and 51. (**C**) Competition of anti-PSGL-1 antibodies 20I15, 18F02, and 19L04 with P-selectin and L-selectin. Serially diluted anti-PSGL-1 antibody was incubated with human PSGL-1-Fc-coated plates, and then biotinylated P-selectin or L-selectin was added at their ~EC50 of binding to PSGL-1 and detected with streptavidin-HRP. Experiments were performed at pH 7.4. (**D**) Competition of anti-PSGL-1 antibodies 20I15, 18F02, and 19L04 with VISTA. Serially diluted anti-PSGL-1 antibody was incubated with human PSGL-1-Fc-coated plates, and then biotinylated VISTA was added at its ~EC50 of binding to PSGL-1 and detected with streptavidin-HRP. Experiments were performed at pH 6.0.

PSGL-1 is a transmembrane mucin-like receptor exclusively expressed in cells of hematopoietic lineage (Figure 1A). Functions of PSGL-1 include its role as an adhesion molecule involved in immune cell trafficking [12,13] and as a regulator of T-cell function [14,15,16,17]. The role of PSGL-1 in modulating the immune function of monocytes, macrophages, and dendritic cells has also been described but less studied [14,18,19,20]. As PSGL-1 has been known to be mainly involved in leukocyte migration and cytokine secretion, it was positioned for the role of promoting immune responses [16]. Recently, we and others demonstrated that PSGL-1 can be targeted to directly stimulate antitumor immunity [15,21,22,23].

As previously reported, PSGL-1 is a macrophage checkpoint expressed across a broad range of solid tumor types and is highly expressed on TAMs and other suppressive myeloid populations [21]. By reanalyzing data from patients treated with a PD-1 inhibitor [24], PD-1 blockade induces upregulation of PSGL-1 expression (Supplementary Figure S1). Given the presence of TAMs across most tumors and their suppressive potential, which is enhanced by PD-1 inhibition, therapeutic strategies have started to incorporate macrophage modulators to drive macrophage repolarization from an M2-like (suppressive) to an M1-like (proinflammatory) phenotype [9,25]. These approaches offer significant clinical potential across most solid tumors, with PSGL-1 being a well-positioned target for this type of intervention. We have recently shown that inhibiting PSGL-1 leads to the repolarization of suppressive M2-like towards an M1 pro-inflammatory macrophages demonstrating that this novel macrophage checkpoint plays a role in maintaining a suppressive functional macrophage state [21].

Here we present preclinical data for the first-in-class PSGL-1 high-affinity inhibitory antibody VTX-0811, which binds to PSGL-1 at a site that does not prevent Selectin or VISTA interaction. VTX-0811 phenocopies siRNA inhibition of PSGL-1 on human macrophages by blocking the PSGL-1-mediated suppression of macrophage functions. VTX-0811 redirects both the macrophages and the tumor microenvironment from a suppressive to an anti-tumor immune response leading to tumor growth inhibition. Furthermore, we present the safety and efficacy data that led to successful IND clearance with the FDA and support the progression of PSGL-1 evaluation in clinical studies.

## 2. Materials and Methods

### 2.1. Antibody Generation and Selection

Anti-PSGL-1 antibodies were generated by mouse immunizations via standard hybridoma procedures by methods described below (LakePharma; Belmont, CA, USA). All immunization work involving animals has been performed under the institutional oversight of LakePharma according to AAALAC animal welfare guidelines. A cohort of eight 6- to 8-week-old mice (CD-1, B6;129, and NZB/w strains) were immunized with recombinant human PSGL-1 Fc fusion protein (hPSGL-1-Fc; R&D Systems, Minneapolis, MN, USA, Cat. 3345-PS; GenBank Accession AAC50061.1), consisting of amino acids Q42-V295 of the human PSGL-1 extracellular domain fused to human IgG1 Fc, and boosted with a final boost of HL-60 cells (ATCC, CCL-240), which endogenously express human PSGL-1 on their surface [26]. Similar cohorts were immunized with a DNA expression vector encoding full-length human PSGL-1, as well as a peptide consisting of the N-terminal 19 amino acids of human PSGL-1 (amino acids 42–60) conjugated to KLH.

The popliteal, iliac, and inguinal lymph nodes and spleens were collected from immunized mice and combined, B-cell-enriched lymphocytes were electrofused with murine myeloma B cells, and the resulting hybridomas were plated at an average of 0.6 cells per well in five 384-well plates. In a primary screen, supernatants from the 384-well plates were evaluated for binding to HEK293T cells stably expressing human PSGL-1 (hPSGL-1-293T) and HL-60 cells (that endogenously express PSGL-1) by flow cytometry, and hybridomas from target positive wells were transferred to 24- or 48-well plates for further expansion. In a confirmatory screen, supernatants from the 24- or 48-well plates were re-assayed for binding to hPSGL-1-293T and HL-60 cells, and target positive wells were further expanded and cryopreserved after the collection of the supernatants. In a final tertiary screen, supernatants were assayed for binding to recombinant His-tagged human PSGL-1 extracellular domain (hPSGL-1-His) and recombinant cynomolgus monkey His-tagged PSGL-1 extracellular domain (cPSGL-1-His) by ELISA, as well as for binding to hPSGL-1-293T cells, parental 293T cells, and HL-60 cells by flow cytometry.

Hybridomas that bound human PSGL-1 expressed on cells and recombinant protein were subcloned by standard limiting dilution methods, and hybridoma supernatants were assessed for binding to hPSGL-1-His by ELISA. Two subclones, A and B, were generated for each hybridoma. Supernatants from 19L04 subclones A and B were confirmed to bind human PSGL-1 as described above. All subsequent studies were performed with the 19L04 hybridoma subclone A. Following subcloning, 19L04 was purified from 100 mL hybridoma supernatant by Protein G affinity chromatography and eluted at pH 3.5. Purified antibody was formulated in 200 mM HEPES, 100 mM sodium chloride, 50 mM sodium acetate, pH 7.0, filtered through a 0.2 µm filter, aliquoted, and frozen. 19L04 was sequenced by standard rapid amplification of cDNA ends (RACE) methods. Recombinant anti-PSGL-1 antibodies were produced in HEK293 cells at ATUM (Newark, CA, USA).

### 2.2. Antibody Humanization

Humanization designs were generated by CDR grafting of the murine 19L04 CDRs into high-identity human germline acceptor frameworks, followed by back-mutation of framework positions to murine 19L04 residues predicted to be structurally important for maintaining CDR loop conformation and binding to PSGL-1. The human variable heavy chain framework IGHV1-2x02 (IgG4, S228P) and the human variable light chain framework IGKV4-1x01 (Igκ) were chosen as acceptor frameworks based upon identity to murine 19L04. The 19L04 CDRs were grafted into these frameworks, and framework positions were reverted to the murine 19L04 sequence based on their potential importance in maintaining 19L04 CDR loop conformation. This humanized antibody is referred to as VTX-0811.

### 2.3. PSGL-1 ELISA

Experiments were performed with plate-immobilized hPSGL-1-His or PSGL-1-Fc protein. PSGL-1 was immobilized on half area plates (Corning, Corning, NY, USA, Cat #3690) at a concentration of 2 µg/mL (25 µL per well) in phosphate-buffered saline (PBS), pH 7.4 (Fisher Scientific, Cat # BP665-1) and incubated for 2 h at 37 °C. Plates were washed 3 times (150 µL per well) with wash buffer (PBS containing 0.05% Tween 20, pH 7.4) and then blocked (150 µL per well) with blocking buffer (3% bovine serum albumin (BSA) in PBS, pH 7.4) for 1 h at 37 °C. Plates were washed 3 times with wash buffer, and antibodies diluted in assay buffer (3% BSA in PBS containing 0.05% Tween 20, pH 7.4) were added (50 µL per well) into appropriate wells. The plates were incubated for 45 min at 37 °C, washed 3 times in wash buffer, and HRP-conjugated anti-mouse IgG (Jackson ImmunoResearch, Cat #115-035-071) or anti-human IgG4 (Invitrogen, Carlsbad, CA, USA, Cat #A10654) antibody added (50 µL per well) for 45 min incubation at 37 °C. Plates were washed 3 times in a wash buffer. Plates were developed with 3,3′,5,5′-tetramethylbenzidine (TMB) HRP substrate (50 µL per well) (Seracare Life Sciences Inc., Milford, MA, USA, cat # 5120-0077) at room temperature for approximately 5 min until sufficient color change was noted. The HRP-TMB reaction was stopped with 1 M sulfuric acid (50 µL per well). Plates were read on the BioTek Cytation 5 instrument at an absorbance of 450 nm.

PSGL-1 peptides were synthesized with a C-terminal biotin (Biomer Technologies, Pleasanton, CA, USA), and ELISAs performed via capture on streptavidin-immobilized half-area plates, according to the conditions described above. The peptides used in the studies are as follows, where Y(SO_3_) denotes sulfated tyrosine:

PSGL-1(42-62)-unmodified: QATEYEYLDYDFLPETEPPEMGGGK-Biotin

PSGL-1(42-62)-sulfated: QATEY(SO_3_)EY(SO_3_)LDY(SO_3_)DFLPETEPPEMGGGK-Biotin.

### 2.4. Ligand Competition ELISAs

Human PSGL-1, Fc chimera (R&D Systems) was immobilized on half-area 96-well plates (Costar^TM^, Thermo Fisher, Waltham, MA, USA) at a concentration of 2 µg/mL (25 µL per well) in Dulbecco’s phosphate-buffered saline (DPBS) overnight at 4 °C. Plates were washed 3 times with 150 µL/well of wash buffer (50 mM HEPES, 125 mM NaCl, 1 mM CaCl_2_, containing 0.05% Tween 20, pH 7.4), then blocked with 150 µL/well of 3% bovine serum albumin (BSA; Thermo Fisher, Waltham, MA USA) in 50 mM HEPES, 125 mM NaCl, 1 mM CaCl_2_, pH 7.4 for 1 h at 37 °C, and then washed 3 times with wash buffer as described above. Anti-PSGL-1 antibodies and human IgG4 isotype control antibodies were titrated 2-fold from 100 nM to 0.1 nM to generate 11-point dose–response curves for each antibody in ELISA buffer (3% BSA in 50 mM HEPES, 125 mM NaCl, 1 mM CaCl_2_, and 0.05% Tween 20, pH 7.4). Antibodies (25 µL/well) were added to PSGL-1-coated plates in duplicate and incubated for 45 min at 37 °C. Biotinylated P- and L-selectin (R&D Systems) (25 µL per well) were then directly added to the plates at 2x their EC50 values (2 nM and 80 nM, respectively, to a final concentration of 1 nM and 40 nM, respectively) and plates incubated for another 45 min at 37 °C. Plates were washed as described previously with 150 µL per well wash buffer. The secondary antibody, Streptavidin HRP conjugate (Jackson ImmunoResearch, West Grove, PA, USA), was diluted 1:1000 in ELISA buffer for a final concentration of 1 µg/mL. A secondary antibody (25 µL/well) was added, and the plates were incubated for 45 min at 37 °C, followed by a final 3 washes with 150 µL/well wash buffer. Plates were developed with 25 µL/well of 3,3′,5,5′-Tetramethylbenzidine (TMB) substrate (SeraCare, Seracare Life Sciences Inc., Milford, MA, USA) at room temperature (RT) for approximately 5 min until sufficient color change was noted. The HRP-TMB reaction was stopped with 25 µL/well of 1 N hydrochloric acid (Thermo Fisher, Waltham, MA USA). Plates were read on the BioTek Cytation 5 instrument at an absorbance of 450 nm. A similar assay protocol was used for VISTA competition, using biotinylated VISTA (R&D Systems, Minneapolis, MN, USA), but substituting the following buffers to perform the assay pH 6.0: wash buffer (25 mM ACES, 150 mM NaCl, 1 mM CaCl_2_, containing 0.05% Tween 20, pH 6.0); blocking buffer (3% BSA in 25 mM ACES, 150 mM NaCl, 1 mM CaCl_2_, pH 6.0); ELISA buffer (3% BSA in 25 mM ACES, 150 mM NaCl, 1 mM CaCl_2_, and 0.05% Tween 20, pH 6.0).

### 2.5. Binding of VTX-0811 to PSGL-1 Expressing and PSGL-1 Knock-Out Cells

The binding of VTX-0811 and isotype control antibodies to HL-60 (ATCC, cat#CCL-240), a PSGL-1-expressing cell line, and to a HL-60 PSGL-1 CRISPR knock-out (Synthego, Redwood City, CA, USA) cell pool was evaluated using flow cytometry. HL-60 or PSGL-1 CRISPR knock-out HL-60 cells were washed twice in flow cytometry (FC) buffer (Dulbecco’s phosphate-buffered saline (DPBS; Gibco/Thermo Fisher, Waltham, MA USA), 5% fetal bovine serum (FBS; Bio Fluid Technologies, Bryn Mawr, PA, USA), 0.05% sodium azide (Ricca Chemical Company, Arlington, TX, USA)), and 50,000 cells/well (at a density of 1 × 10^6^ cells/mL) were plated in a 96 well plate. Antibodies were diluted from a starting concentration of 120 nM to 0.16 nM in 3-fold dilutions in FC buffer. Plated cells were spun at 500× *g* (RCF) for 3 min and the supernatant was decanted. Antibody dilutions were added to the plated cells (50 mL/well), in duplicate. Plates were covered and incubated for 1 h at 4 °C. After incubation, plates were washed with 100 µL FC buffer. The secondary antibody, mouse huIgG-PE conjugate (Abcam, Waltham, MA, USA), was diluted 1:500 in FC buffer and 50 mL was added to each well. Plates were incubated, protected from light, for 30 min at 4 °C. Plates were washed twice with 100 µL of FC buffer. Fixation buffer (100 µL; 1% Paraformaldehyde (Alfa Aesar, Haverhill, MA, USA) in FC buffer) was added to each well. The plate was covered and protected from light until analysis. Plates were read on the Attune NxT flow cytometer attached to the Attune autosampler to generate geometric mean fluorescent intensity (gMFI) values.

### 2.6. Macrophage Differentiation and Polarization

Human PBMC samples or whole-blood Leukopaks were purchased from Research Blood Components, LLC. (Watertown, MA, USA). Research Blood Components follows the American Association of Blood Banks guidelines for drawing donors. The donor population consisted of healthy males and females between the ages of 18 and 65. All donors completed a uniform blood donor history questionnaire. An IRB-approved consent form was obtained from each donor giving permission to collect their blood and use or sell it at the Research Blood Components discretion for research purposes. Confidentiality and donor identification were assured. For more information, please see https://www.researchbloodcomponents.com/about-us (accessed on 30 May 2022).

PBMCs were isolated from leukopaks as per standard methods such as (Protocol for Leukopak Processing and Washing for Downstream Cell Isolation|STEMCELL Technologies). Monocytes were enriched from the PBMCs using a Stemcell monocyte negative selection kit (Stemcell Technologies, Vancouver, BC, Canada, Cat # 19058) following the manufacturer’s recommendation. Enriched monocytes were centrifuged at 300× *g* for 5 min at RT, and the cell pellet was resuspended in assay media (Iscove’s Modified Dulbecco’s Medium (IMDM) containing 10% FBS) to a final concentration of 4 × 10^5^ cells/mL.

To differentiate and polarize monocytes into M2c macrophage, enriched monocytes from each donor (5 donors total; 1 mL of 4 × 10^5^ cells/mL per well) were added to six 24-well plates and incubated at 37 °C overnight (Day 0). Twenty-four hours after plating monocytes (Day 1), the media was aspirated and 1 mL of fresh assay media containing 50 ng/mL human macrophage colony-stimulating factor (M-CSF; Biolegend, San Diego, CA, USA, Cat #574804) was added per well. The cells were incubated at 37 °C for 72 h. On Day 4, 500 µL of assay media was removed and replaced with 500 µL fresh assay media containing M-CSF, and the cells were incubated at 37 °C for an additional 48 h. On Day 6 of the assay, differentiated M2 cells were polarized to M2c macrophages. Media was removed from cells and replaced with 1 mL of assay media containing 50 ng/mL M-CSF and 10 ng/mL of interleukin-10 (IL-10, Biolegend, San Diego, CA, USA, Cat #574004), and the cells were then incubated at 37 °C for 24 h.

### 2.7. Macrophage Functional Assay

To evaluate the effect of 19L04c on M2c function, on Day 7, M2c macrophages from 6 donors were treated with 19L04c or isotype control at 10 µg/mL in the 24-well format for 30 min at 37 °C. The cells were then activated with 100 ng/mL LPS (InvivoGen, San Diego, CA, USA, Cat #tlrl-eblps) in assay media containing 50 ng/mL M-CSF and 10 ng/mL IL-10 and further incubated at 37 °C for 24 h. After the incubation period, the plates were centrifuged at 500× *g* for 5 min at RT, and the supernatants were collected and stored at −80 °C until assessment for mediator production using a cytokine 25-plex human Luminex panel (Invitrogen, Carlsbad, CA, USA, Cat #LHC0009M).

### 2.8. Functionality in Multi-Cellular Assays

To demonstrate that VTX-0811 activity on macrophages can be translated into a coordinated immune response within a more complex system, two assays were established. In the first assay, human PBMCs were stimulated with Staphylococcus Enterotoxin Type B (SEB), which cross-links MHC-II expressed on antigen-presenting cells with the T-cell receptor on T cells. For the second assay, we established a mixed lymphocyte response assay using M2 macrophages and allogeneic T cells.

For the SEB-PBMC assay, 2 × 10^5^ PBMCs (100 µL) in complete media (RPMI containing 10% fetal bovine serum (FBS), 1X non-essential amino acids, 1 mM sodium pyruvate, 10 mM HEPES and 0.1% 2-mercaptoethanol) were aliquoted per well into a 96-well plate. 19L04c and VTX-0811 (50 µL/well) were added to PBMCs at final concentrations ranging from 60 µg/mL to 9.1 ng/mL. Complete RPMI media (50 µL/well) was also added to each well, and plates were then incubated at 37 °C for 15 min. SEB (Millipore Sigma, Burlington, MA, USA, 324798) was then added at 0.25 µg/mL final concentration per well, a pre-determined sub-optimal concentration that stimulated PBMCs without inducing a maximal stimulatory response. The plate was then incubated at 37 °C for 4 days. Following incubation, the plate was centrifuged at 350× *g* for 5 min at RT, and the culture supernatants were collected and stored at −80 °C until being analyzed for secreted mediators by Luminex and ELISA (chemokine (C-C motif) ligands 4 (CCL4)).

For the MLR assay, on Day 1, monocytes were isolated as described above, adjusted to 500,000 cells/mL in IMDM + 10% FBS, and 100 µL (50,000 cells) was added per well of a 96-well flat bottom plate. Twenty-four hours later (Day 2), there was a full media change with 100 µL of M0 media (IMDM + 10% FBS + 50 ng/mL M-CSF) followed by the addition of 100 µL of M0 media with 20 µg/mL 19L04c or the isotype control (10 µg/mL final concentration). On Day 5 and Day 7, there was a half media change with 100 µL of M0 media containing 10 µg/mL mAb (10 µg/mL final concentration). On Day 9, T cells were isolated from allogeneic donor PBMCs using a total CD3-negative isolation kit (Stemcell Technologies, EasySep™ Human T Cell Isolation Kit). The T cells were washed 1X with 200 µL of warm T-cell media (RMPI 1640 containing 10% FBS, 1X NEAA, 1 mM sodium pyruvate, 10 mM HEPES, and freshly added 55 µM 2βME (beta-mercaptoethanol)), resuspended at 1 × 10^6^ cells/mL, and 100 µL of T cells were added per well to the M0 macrophages. The MLR reaction was incubated for 4 days and on Day 13, supernatant was collected for Luminex analysis of mediators, and the cells were processed for flow cytometry.

### 2.9. NSG-SGM3-BLT Humanized Tumor-Bearing Mouse Model

All experiments were performed in accordance with the guidelines of the Institutional Animal Care and Use Committee of the University of Massachusetts Medical School and the recommendations in the Guide for the Care and Use of Laboratory Animals (Institute of Laboratory Animal Resources, National Research Council, National Academy of Sciences, 1996).

To assess the effect of 19L04 on tumor growth, anti-human PSGL-1 19L04c antibody was evaluated in a humanized NSG-SGM3-BLT mouse model of melanoma. The model was set up as previously described [21]. In brief, 10-week-old NSG-SGM3 mice (Jackson Laboratories) were engrafted with fragments of human fetal liver and thymus under the mouse kidney capsule. Two weeks later, the mice were irradiated (100 cGy gamma) to destroy the mouse bone marrow and humanized with 2 × 10^5^ human CD34^+^ hematopoietic progenitor cells (HSC) to reconstitute a human immune system. The degree of humanization was assessed 8 weeks post-humanization with CD34+ HSCs by performing flow cytometry on whole blood (200 µL) with anti-mouse CD45, anti-human CD45, anti-human CD3, and anti-human CD20 antibodies. Mice were considered well-humanized and ready for studies when the degree of humanization (human CD45+ cells) was approximately 20–30% of all immune cells. All mice had quantifiable T-cell (12–95% of CD45 population) and B-cell (0.6–46% of CD45 population) populations in serum indicating maturation of human lymphocytes in the model.

At 6 weeks post HSC injection, mice were inoculated with patient-derived xenograft (PDX) melanoma cells (AV17.26) and randomized into groups after tumors grew to a palpable size of approximately 50–100 mm^3^. Mice were administered 10 mg/kg of 19L04c or Isotype (negative control antibody), while anti-PD-1 (Pembrolizumab) was dosed at an initial dose of 10 mg/kg and all subsequent doses at 5 mg/kg—a regimen optimized by M. Brehm and colleagues in previous experiments (personal communication). Mice were dosed twice weekly for 3.5 weeks, tumor volumes were measured using calipers, and following the last dose mice were euthanized, and tumors were harvested to evaluate infiltrating immune cell composition.

### 2.10. Flow Cytometry Analysis of Tumors

Explanted tumors were mechanically dissociated by chopping and grinding tumor fragments through mesh screens followed by passing through cell strainers. About 1 mL of 500 × 10^6^ live single-cell suspension cells/mL was stained for viability using Fixable Viability Dye eFluor780 (eBioscience/Thermo Fisher, Waltham, MA USA), followed by blocking unspecific antibody binding by incubating in 1 mL Fc-blocking buffer (human FcX; Biolegend, San Diego, CA, USA) for 10 min. Following the blocking step, 100 µL of cells were combined with 100 µL of the lymphoid or myeloid staining cocktail and incubated for 1 h on ice. The lymphoid staining cocktail contained CD3, CD4, CD8, CD16, CD20, CD25, CD45, CD45RA, CD56, MHCI, PD-1, and PSGL-1. The myeloid staining cocktail contained CD3, CD11b, CD14, CD16, CD33, CD45, CD86, CD163, CD206, CSF1R (CD115), HLA-DR (MHCII), PSGL-1, and VSIG4. Stained cells were resuspended in a fixation buffer containing 0.32% paraformaldehyde (ThermoFisher Scientific). Fixed samples were acquired using an AttuneTM NxT Flow Cytometer using AttuneTM NxT Software (Version 4.2.0) for acquisition. Data were analyzed using FlowJo software version 10.7.1 (Becton Dickinson & Company, Franklin Lakes, NJ, USA). Distinct macrophage populations were defined as CD45^+^ CD33^+^ or CD14^+^CD11b^+^ gated from CD45^+^ viable cells. Macrophage populations were then analyzed for the presence of M1 phenotypic markers (MHCII and CD86) or M2 phenotypic markers (CD163 or CD206).

For multivariate analysis of flow cytometric data (UMAP), acquisition files in Flow Cytometry Standard (FCS) format 3.1 were exported from the flow cytometer and analyzed in the R statistical computing environment (v4.0.3). The flowCore (v2.3.1; 9) R package was used to import, compensate, and transform FCS files. For data transformation, a special class of biexponential functions known as the logicle transformation was used. Marginal events, debris, and doublets were removed using the “openCyto” (v2.2.0; 10) and “flowDensity” (v1.24.0; 11) R packages. Marginal events were defined using forward scatter (FSC) FSC-A and side scatter (SSC) SSC-A parameters. Debris was defined using FSC-A. Doublets were defined using FSC-A and FSC-W. CD45^+^ immune cells were gated based on the “VL3-A CD45-A” (BV605) channel for each sample, then pooled across all samples for downstream multivariate analysis. Analysis of CD45^+^ Tumor-Infiltrating Immune Cells Algorithms based on clustering, dimensionality reduction, and trajectory inference fully switch from the univariate/bivariate analysis to a multivariate approach. These tools consider the distribution of all markers simultaneously in the whole dataset, overcoming many manual gating limitations. Prior to dimensionality reduction, forward and side scatter parameters (FSC-A, FSC-H, FSC-W, SSC-A, SSC-H, SSC-W) were normalized using the z-score method. Dimensionality reduction using the UMAP (Uniform Manifold Approximation and Projection) algorithm was performed on a 20-dimensional data matrix (3 FSC parameters, 3 SSC parameters, 14 fluorescent markers). The “umap” (v 0.2.7.0) R package was used with the default settings. Manual gating analysis was performed on dimensional-reduced 2D UMAP maps, and phenotypically complex gated cell populations were further subjected to UMAP projection and manual gating. For each unique cell population per sample, the mean fluorescence intensity of each marker was calculated as the arithmetic mean of the logicle-transformed fluorescence intensities. Statistical analyses were performed on tumor volumes at the termination of the study using GraphPad Prism (v9.1.0). Tumor volumes from different groups were compared using two-way ANOVA tests with multiple comparisons using Bonferroni correction.

### 2.11. Ex Vivo Patient-Derived Human Tumor Cultures

Fresh human tumor tissue originating from the discarded sample was sent on wet ice in DMEM within no more than 24 h after surgery for culture. Tissue samples were provided by the NCI Cooperative Human Tissue Network (CHTN, https://chtn.cancer.gov/, accessed on 13 June 2022), the National Disease Research Interchange (NDRI, https://ndriresource.org/, accessed on 13 June 2022), or BioIVT (https://bioivt.com/, accessed on 13 June 2022). Tissue samples were obtained using all the applicable operating policies, and procedures that protect the subjects from whom specimens are obtained. These policies and procedures are consistent with current regulations and guidance for repositories from the Office of Human Research Protections (OHRP, DHHS).

Eleven fresh primary human tumors (5 kidney, 3 uterine, 1 endometrial, 1 ovarian, and 1 omentum) were dissociated using enzymatic digestion and cultured in the presence of 19L04c, Pembrolizumab, or isotype control antibodies for 48 h. Fresh tumor tissue was placed in DMEM within 60 min of surgical resection and moved to a tissue culture-treated petri dish containing 20 mL of cold Hanks balanced salt solution (HBSS; Gibco). After removing fat, fibrous, and necrotic areas, tumors were cut into small pieces of 2–4 mm and subsequently transferred into the MACS enzyme mix and tumors were further minced. The dissociation enzyme mix was prepared from a MACS tumor dissociation kit (Miltenyi Biotec, Gaithersburg, MD, USA) by adding 200 µL of Enzyme H, 100 µL of Enzyme R, and 25 µL Enzyme A to 4.7 mL of DMEM. Samples were vortexed and incubated at 37 °C for 45 min to 1 h. Digested tumors were filtered with 40 µm cell strainers and subsequently incubated in ice-cold DMEM supplemented with 8% FBS, 2% human serum, 100 IU/mL penicillin/streptomycin, 1 mM Glutamax, 55 µM 2-ME, 1X non-essential amino acids, 1 mM sodium pyruvate, 48 ng/mL human recombinant IL-2 (100 IU/mL, assuming a specific activity of 2.1 × 10^6^ IU/mg), 1X insulin/transferrin/selenium, and 4 ng/mL human M-CSF to stop the enzymatic reaction. After centrifugation at 300× *g*, for 5 min at RT, cell pellets were resuspended in a culture medium and the cells were counted. About 5 × 10^5^ cells/mL were plated/well into 6-well plates and incubated with 10 µg/mL of each antibody (anti-PSGL-1, isotype control, and pembrolizumab) for 48 h. Supernatants from the dissociated tumor cultures were analyzed using a cytokine 25-plex human Luminex panel (Invitrogen) according to the manufacturer’s instructions. Cytokine levels for the duplicate incubations with 19L04c, Isotype, and Pembrolizumab were averaged. The treatment effect was then assessed as percent induction of the treatment arms (19L04c or Pembrolizumab) over the isotype control arm normalized to the isotype control using the following formula:

Treatment Effect (% induction) = ((Treatment – Isotype)/Isotype) × 100%

### 2.12. NHP Pharmacokinetic and Toxicology Studies

PK was evaluated in monkeys treated with a single dose of VTX-0811. Eighteen naïve cynomolgus monkeys were randomly assigned to 3 groups of 3 females and 3 males per group. VTX-0811 was dosed at 3, 10, and 30 mg/kg once via 10 min intravenous (iv) infusion at a dose volume of 3 mL/kg. Blood samples for PK analysis were collected at pre-dose, 0.17 h (10 min), 6 h, 24 h, 48 h, 72 h, 96 h, 168 h, 264 h, 360 h, 456 h, 552 h, and 648 h post-dose.

Next, a 4-week toxicity and toxicokinetic study of VTX-0811 was performed. VTX-0811 was administered once weekly for 4 consecutive weeks (on Days 1, 8, 15, 22, and 29) via i.v. infusion (30 min at a dose volume of 10 mL/kg) to investigate the reversibility, progression, and/or potential delayed effects during a 4-week recovery period. Naïve monkeys (n = 40) were randomly assigned to 4 groups at 0, 25, 75, and 200 mg/kg/dose. Each group had 5 F and 5 M monkeys with 3 F and 3 M monkeys in each group being sacrificed on Day 30, while 2 F and 2 M monkeys from each group were maintained through the recovery phase, being sacrificed on Day 58 following a 29-day untreated period.

To assess the RO levels of VTX-0811 on peripheral cells after VTX-0811 treatment, a qualified RO method (MQ 99205-201251) was used to determine the RO of VTX-0811 binding to PSGL-1 on the surface of T cells in whole blood in terms of antibody-binding capacity.

Parameters for toxicity evaluations included mortality, clinical observations, body weights and body weight changes, food consumption (qualitative), Draize evaluation at the injection site, body temperature, blood pressure, electrocardiogram (ECG), ophthalmology, clinical pathology (clinical chemistry, hematology, coagulation, and urinalysis), absolute and relative organ weights, gross pathology, and histopathology evaluation.

Blood samples for TK were collected during Week 1, on Day 15, and during Week 4; for cytokine analysis once prior to the first dosing, 2 h post first dosing, 24 h post first dosing, 2 h post last dosing, and on Day 58; for complement (C3 and C4) and globulin (IgA, IgG, and IgM) analysis once prior to the first dosing, 24 h post first dosing and prior to necropsy; for immunophenotyping once prior to the first dosing, 24 h post first dosing and prior to necropsy.

Immunophenotyping was assessed by flow cytometry (FCM). The percentages and absolute numbers of lymphocyte subpopulations of total T cells (CD45^+^CD16^–^D3^+^), cytotoxic T (Tc) cells (CD45^+^CD16^–^CD3^+^CD4^–^CD8^+^), double-positive (DP) T cells (CD45^+^CD16^–^CD3^+^CD4^+^CD8^+^), double-negative (DN) T cells (CD45^+^CD16^–^CD3^+^CD4^–^CD8^–^), helper T cells (CD45^+^CD16^–^CD3^+^CD4^+^CD8^–^), B cells (CD45^+^CD16^–^CD20^+^), and NK cells (CD45^+^CD3^–^CD16^+^) in the peripheral blood from cynomolgus monkeys.

This study was conducted in compliance with U.S. FDA Good Laboratory Practice (GLP) Regulations for Nonclinical Laboratory Studies (21 CFR Part 58) and National Medical Products Administration (NMPA) Good Laboratory Practice (GLP), No. 34 (1 September 2017). The characterization of the test article was analyzed under non-GMP conditions. The manufacture of the Test Article and Control Article, VTX-0811 injection, and VTX-0811 injection placebo, respectively, were GMP-compliant.

### 2.13. Naïve Unstimulated PBMCs Assay

The assay was based on features identified to improve assay sensitivity [27,28,29] for the human CD28 superagonist, TGN1412. Based on these modifications, the agonist potential of VTX-0811 was evaluated as an immobilized or soluble antibody using short-term pre-cultured PBMCs. TGN1412 was used as a positive control in both assay formats, and an isotype control IgG4 antibody (ATUM-produced control anti-respiratory syncytial virus (RSV) antibody based on the palivizumab sequence on an IgG4 backbone) was included as a negative control for VTX-0811.

To prepare the pre-cultured PBMCs, 200 µL (2 × 10^5^ cells) of the PBMCs were aliquoted per well into a 96-well round bottom tissue culture plate, and the cells were cultured for 48 h at 37 °C. After incubation, on Day 3, the PBMCs were harvested, washed with Dulbecco’s phosphate-buffered saline (DPBS), centrifuged at 350× *g* for 5 min at RT, and resuspended at 1 × 10^7^ cells/mL in complete RPMI media.

For the soluble assay format, on Day 3, 100 µL (1 × 10^6^ cells) of the pre-cultured PBMCs was aliquoted per well in triplicate into a 96-well round bottom plate. VTX-0811 and the IgG4 isotype control antibody were diluted to 2× the final concentrations of 1000, 10, 1, 0.1, and 0.01 mg/mL, and the positive control anti-CD28 antibody was diluted to 2× the final concentrations of 50, 10, and 1 µg/mL. All antibodies were diluted in complete RPMI media, and 100 µL per well of the 2× concentration of each antibody was added to the PBMCs with a final volume in the plate of 200 µL/well. The cells were incubated at 37 °C/5%CO_2_ for 48 h.

For the plate-bound assay format, on Day 2, tissue culture treated, flat bottom, 96-well plates were coated with 100 µL/well of F(ab′)2 goat anti-human IgG, IgM (H + L) (Invitrogen, Carlsbad, CA, USA, Cat #16-5099-85) in DPBS at a final concentration of 10 µg/mL, and the plates were incubated at 4 °C overnight. After incubation, on Day 3, the F(ab′)2 goat anti-human IgG, IgM (H + L) coated plates were washed twice with 200 µL/well DPBS; 100 µL per well of VTX-0811 and the IgG4 isotype control antibody were added at final concentrations of 1000, 10, 1, 0.1, and 0.01 mg/mL, and the TGN1412 antibody was added at final concentrations of 50, 10, and 1 µg/mL (100 µL/well). All antibodies were diluted in complete RPMI media. The plates were incubated at 37 °C/5% CO_2_ for 2 h, and then 100 µL (1 × 10^6^ cells) per well of the pre-cultured PBMCs was then added into the antibody-coated, and further incubated at 37 °C for 48 h.

For both assay formats, on Day 5, the cells were centrifuged at 500× *g* for 5 min at RT, and the supernatants were collected into new 96-well plates and stored at −80 °C until analysis of cytokine levels using a cytokine 25-plex human Luminex panel (Invitrogen, Carlsbad, CA, USA, Cat #LHC0009M). Raw data were generated in FlexMap3D software (Updated in May, 2021) and then exported to Microsoft Excel (Version 2205). Compiled data graphs were then generated in GraphPad Prism for final analysis.

### 2.14. CD3-Stimulated T-Cell Assay

Tissue culture plates were pre-coated with 5 µg/mL anti-CD3 (Thermo Fisher Scientific, Cat #16-0031-82) in PBS at 4 °C overnight, then removed. T cells were added at a density of 5 × 10^5^/mL in 10% FCS RPMI medium and incubated at 37 degrees for 3 days with anti-CD28 antibody at 5 µg/mL (Thermo Fisher Scientific, Waltham, MA, USA Cat # 16-0289-81). 19L04c or the isotype control was used at 10 µg/mL final concentration and added to the culture with an anti-CD28 antibody. At the end of 3 days, cells were stained for analysis by flow cytometry, and supernatants were collected to be analyzed by ELISA. For flow cytometry, the cells were labeled with a viability dye (Fixable Viability Dye eFluor780 (eBioscience/Thermo Fisher Scientific, Waltham, MA, USA) as per the manufacturer’s recommendation), washed in FACS buffer, and 5 µL of FcX human blocking buffer (5 µL per well, BioLegend, San Diego, CA, USA Cat # 422301) was added per well for 10 min at RT. This step was followed by the addition of 50 µL of the staining panel (CD45, CD3, CD8, and CD69, BioLegend, San Diego, CA, USA). The cells were then washed twice with FACS buffer, resuspended in FACS buffer, and the data were acquired on the Attune flow cytometer. Data are presented as the % CD69+CD8+ T cells from viable total CD3 cells.

### 2.15. Phagocytosis Assay

To evaluate the effect of anti-PSGL-1 blockade on phagocytosis, M2c macrophages were treated with 19L04c prior to co-incubation with labeled tumor cells. Human M2c macrophages were generated as described above.

SK-MEL-5 was plated in the RMPI/10% FBS on Day 1 and split on Day 3 into multiple T75 flasks and grown to 80–90% confluency in the flasks. Non-adherent cells were removed by washing the cells with 5 mL of PBS, and adherent cells were detached from the plastic with Trypsin/EDTA, transferred to a 50 mL conical, centrifuged for 5 min at 350× *g* at RT, washed with RMPI/10% FBS, and resuspended in 5 mL of RMPI/10% FBS. Cells were then counted and labeled with pHrodo Red stain by adding 90 µL of pHrodo stock (prepared as per the manufacturer’s recommendation, ThermoFisher Scientific, Waltham, MA, USA, Cat # P35372) to SK-MEL-5 at 1 × 10^6^ cells/mL for a final concentration of 120 ng/mL and incubating the cells for 30 min on plate rotator at RT. Following the 30 min incubation, 10 mL of PBS was added, the cells were then centrifuged, washed with PBS, and resuspended in 5 mL of RMPI/10% FBS. During the 30 min incubation period of pHrodo Red labeling of the SK-MEL-5 cells, test articles and control antibodies were added to the M2c macrophages at 10 µg/mL in the 24-well differentiation/polarization plates.

For the phagocytosis assay, the treated M2c macrophages were co-cultured with the pHrodo Red labeled SK-MEL-5 cells. About 500 µL of pHrodo-labeled SK-MEL-5 cells at 8 × 10^5^ cells/mL (400,000 cells) was added to the macrophages. The macrophages were not detached and counted after the differentiation/polarization step; therefore, the ratio of tumor cells to macrophages was based on the original 400,000 monocytes per well and in a 500 µL volume to obtain a 1:1 ratio. The M2c/SK-MEL-5 co-culture plate was incubated at 37 °C for 2 h to allow the phagocytosis of the labeled cancer cells.

To evaluate the degree of phagocytosis, the media was removed from wells containing the M2c/SK-MEL-5 cells, and the cells were washed 1× with RMPI/10% FBS per well. The plates were placed on ice to slow/stop phagocytosis, the cells were gently scrapped to release them from the plate, and the cell suspension was pipetted into a 1 mL 96-well deep well plate, centrifuged and resuspended in 200 µL per well of FACS buffer. The cells were then incubated with an FcX blocking buffer (5 µL per well, BioLegend, San Diego, CA, USA, Cat # 422301), labeled with a viability dye (Fixable Viability Dye eFluor780, eBioscience, Cat # 65-0865-14 as per the manufacturer’s recommendation), washed with FACS buffer, and 5 µL of FcX human blocking buffer was added per well for 10 min at RT. This step was followed by the addition of 50 µL of either the myeloid staining panel (CD45, CD86, live/dead-viability dye from BioLegend, San Diego, CA, USA and CD163 from R&D Systems, Minneapolis, MN, USA) or the isotype control or FMO stain and incubated for 1 h on ice. After the incubation, 300 µL of FACS buffer was added per well and the cells were centrifuged, washed with FACS buffer, resuspended in 200 µL of fixation buffer per well, and immediately run on the Attune flow cytometer. Phagocytosis of the cancer cells was measured based on CD163^+^SK-MEL-5^+^ double positivity. CD163 is a marker of M2 macrophages and SK-MEL-5 positivity is measured by the presence of pHrodo dye in CD163^+^ macrophages due to the phagocytosis of the labeled SK-MEL-5 cells.

### 2.16. Neutrophil Activation Assay

Blood from healthy human donors was collected in acid-citrate dextrose (ACD) tubes and processed within 2–3 h of the blood draw. About 490 µL of whole blood was pipetted per well into a 96-deep well plate (ThermoFisher, Waltham, MA, USA, Cat #260252); 5 µL of stimulant or diluted antibody was added per well to the appropriate wells for a total volume of 500 µL. The stimulant fMLP (200 mM stock from Sigma Aldrich, Burlington, MA, USA Cat #F3506) was diluted in PBS and used at a concentration of 0.24 µM, a concentration determined from a previous neutrophil activation assay as the EC80. Antibodies were added at a final concentration of 10 μg/mL, 1 μg/mL, or 0.1 μg/mL. To understand the effect of the antibodies on naïve neutrophils, the cells were treated with the antibodies and incubated for 1 h without stimulation (No stimulation condition). To evaluate the effect of the antibodies on the ability of neutrophils to be activated, the cells were treated with the antibodies for 1 h and then stimulated for 15 min at 37 °C (Pre-stimulation). To evaluate the effect of the antibodies on activated neutrophils, cells were stimulated for 15 min at 37 °C and then treated with the antibodies and incubated at 37 °C for an additional 1 h (Post-stimulation).

At the completion of the assay period, the assay plate was placed on ice to stop the reaction, and the blood suspension was mixed with a 300 µL multi-channel pipette set to 160 µL. About 40 μL of the stimulated whole blood was added to a V-bottom 96-well plate (Corning, Glendale, AZ, USA, Cat. #3797) containing 160 μL of ice-cold neutrophil wash buffer (NWB: 2.5 g BSA (0.25%), 10 mL of Hepes, 1.5% dextrose (~8 mM), brought to liter with 1× PBS without Ca^2+^ and Mg^2+^) for staining. The blood and NWB suspension was mixed by setting a pipette to 50 μL and pipetting up and down 3 times, centrifuged at 1200 rpm for 5 min, discarded supernatant, added 50 μL viability dye (Invitrogen, Waltham, MA, USA, Cat. #65-0865-14, 1:1000), and incubated for 10 min on ice followed by the addition of 200 μL of NWB per well. The plate was centrifuged at 1200 rpm for 5 min, the supernatant was discarded by pipetting, and the pellet was resuspended in 10 μL blocking buffer (Human TruStain FcX Biolegend, San Diego, CA, USA, Cat #422302 diluted 1:20 in FACS staining buffer) and incubated for 10 min on ice. Next, a 60 µL staining cocktail (CD66b, CD45, CD15 CXCR2, CD11b CD63 CD62L) or FMOs or isotype cocktail was added per well, and the plate was incubated for 20 min on ice followed by the addition of 120 μL NWB to each well and gentle mixing. The plate was centrifuged, the supernatant discarded by pipetting, and the cells were washed with another 200 μL NWB. After centrifugation, 100 μL of a secondary antibody (Invitrogen, Waltham, MA, USA, Cat. #A55749; diluted 1:10) was added per well, and the cells were incubated for 10 min on ice followed by the addition of 120 μL NWB to each well. The cells were centrifuged and washed with 200 μL NWB as above and then resuspended in 200 μL of 1× Lysis buffer (Becton Dickinson, Franklin Lakes, NJ, USA, Cat #349202 1:10 in ddH_2_0) and incubated for 10 min at room temperature. The cells were again centrifuged and washed 2× with 200 μL each time of NWB, resuspended in 200 μL of NWB, and run on attune. Data are presented as the MFI of the indicated marker (CD11b, CD62L, or CXCR2) on viable CD66b^+^CD15^+^ neutrophils.

Note that there is no fixation step and that a positive control for cell death was prepared by adding 100 µL of viability dye to a well containing whole blood, and the plate was placed at −20 °C for 10 min and then maintained at RT and subjected to the lysis step as described above.

## 3. Results

### 3.1. Antibody Selection and Humanization

With the aim of blocking the inhibitory role of PSGL-1 in human TAMs and changing the functionality of PSGL-1-expressing TAMs in human tumors, we initiated antibody discovery campaigns for PSGL-1. As we did not want to postulate which part of the PSGL-1 molecule is responsible for the inhibitory myeloid function, we generated PSGL-1-specific antibodies with broad epitope diversity and relied on functional assays to drive the selection of the lead. To develop a diverse antibody panel, mice were immunized with multiple forms of PSGL-1: recombinant full-length PSGL-1 extracellular domain protein; DNA encoding full-length PSGL-1 to allow PSGL-1 to be presented in a more native context; and a peptide corresponding to the PSGL-1 N-terminus conjugated to the carrier keyhole limpet hemocyanin (KLH), to direct antibody generation to the site on the PSGL-1 molecule mediating known ligand interactions with Selectins and VISTA (Figure 1A).

Overall, more than 7500 hybridomas were screened leading to the identification of >100 PSGL-1-specific antibodies, which bound PSGL-1 expressed naturally or on engineered to over-express cell lines. These hybridomas recognized at least six epitopes, including ligand-blocking and non-blocking epitopes within the PSGL-1 N-terminus as well as epitopes outside of the N-terminus. Twenty-six hybridomas were selected for sub-cloning and further biochemical analysis assays, based on their affinity and specificity for human PSGL-1, as well as cross-reactivity to cynomolgus monkey PSGL-1.

Hybridoma-derived antibodies were initially evaluated in vitro in two functional assays to probe whether anti-PSGL-1 antibodies induce inflammatory responses: (i) a SEB-stimulated human PBMC assay, similar to what has been previously used to screen for the effect of antibodies on immune cell activation [30], and (ii) an M2c macrophage assay, to assess the ability of antibodies to repolarize LPS-stimulated M2c macrophages towards an M1 phenotype. Several antibodies induced the secretion of pro-inflammatory mediators in the SEB-PBMC assay as well as induced varying degrees of repolarization of M2c macrophages, as demonstrated by their ability to induce the secretion of the M1-associated mediator GM-CSF (Supplementary Figure S2). To confirm the activity of these antibodies, heavy and light chain variable domains were sequenced from a subset of hybridomas and expressed as chimeric human IgG4 antibodies.

These screens identified anti-PSGL-1 antibodies that drive repolarization of primary human macrophages from three different epitope groups: N-terminal binding with complete blocking of Selectin-PSGL-1 interactions; N-terminal binding with partial blocking of Selectin-PSGL-1 interactions; and non-blocking antibodies that bound outside of the PSGL-1 N-terminus (Figure 1 and Figure S2). Clone 19L04, which induces strong macrophage repolarization and does not interfere with the ability of PSGL-1 to interact with Selectins, was selected as the lead antibody for the program (Figure 1).

19L04 was subsequently humanized by CDR grafting of the murine 19L04 CDRs into high-identity human germline acceptor frameworks, resulting in VTX-0811, which consists of the humanized 19L04 variable regions in a human IgG4 (S228P) backbone. The EC50 values of chimeric 19L04 (termed 19L04c) and VTX-0811 binding to bivalent human PSGL-1 were similar at 1.26 and 1.46 nM, respectively, indicating that humanization of the chimeric antibody did not adversely affect target PSGL-1 binding (Supplementary Figure S3). VTX-0811 binds cell surface PSGL-1 and is specific for PSGL-1, as well as recognizes the cynomolgus monkey ortholog of PSGL-1 (Supplementary Figure S3).

### 3.2. VTX-0811, the Lead Antibody, Induces a Broad Pro-Inflammatory Response

**Figure 2:**
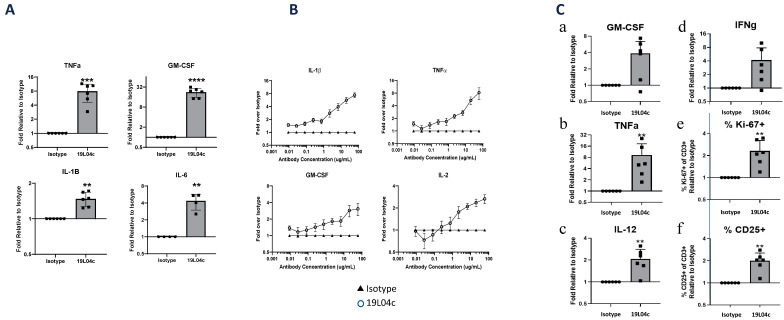
19L04c induced macrophage repolarization and pro-inflammatory activation in multicellular assays. (**A**) M2c repolarization. Fold-change relative to IgG4 isotype for each donor of secreted pro-inflammatory proteins TNFa, IL-6, IL-1b, and GM-CSF measured by Luminex in the supernatants from LPS stimulated M2c macrophages treated with 19L04c (n = 6 for GM-CSF, TNFa, and IL-1b; n = 4 for IL-6). (**B**) SEB-activated PBMC assay. Fold change relative to IgG4 isotype for each donor of secreted pro-inflammatory proteins IL-1b, IL-2, TNFa, and IFNg measured by Luminex in the supernatants from SEB-stimulated PBMCs treated with 19L04c. (n = 6 donors) (**C**) MLR assay. Fold change relative to IgG4 isotype for each donor of secreted proteins from an MLR assay. M0 macrophages were treated with 19L04c throughout the 9-day differentiation and polarization of M0 macrophages. The macrophages were then washed to remove residual 19L04c prior to co-incubation with allogeneic T cells for 4 days. On Day 13, Luminex was used to measure secreted mediators from macrophages (**a**–**c**) and T cells (**d**) in the supernatants, and flow cytometry was used for T-cell proliferation and activation (**e**,**f**). n = 6 donor pairs (three monocyte donors; two T-cell donors). * denotes the levels of significance. *p*-value ≤0.01 (**), ≤0.001 (***), ≤0.0001 (****).

To evaluate the effect of 19L04c on modulating immune functionality, the antibody was evaluated in macrophage only and in multiple cell types containing primary human cell assays. First, LPS stimulation of M2c macrophages pre-incubated with 19L04c led to the secretion of multiple M1-associated mediators including GM-CSF, TNFa, IL-1b, and IL-6 with an average of 22, 8, and 1.5 and 4-fold change, respectively, across 6 donors relative to isotype control antibody (Figure 2A). 19L04c treatment of human PBMCs activated with SEB led to a dose-dependent increase in multiple pro-inflammatory mediators including primarily macrophage-derived IL-1b, TNFa, and GM-CSF. Furthermore, the increase in secreted IL-2 levels supports the effect of anti-PSGL-1-induced macrophage repolarization on T-cell activation (Figure 2B).

In the SEB-PBMC assay, we could not distinguish if 19L04c induced T-cell activation by direct or indirect effect of the antibody on T cells, as multiple cell types in PBMC express PSGL-1 and bind to 19L04c antibody. To attempt to distinguish between these two options, we utilized a mixed lymphocyte (MLR) assay. In this version of the MLR assay, we isolated monocytes and T cells from the same donor. Monocytes were used to differentiate M2c cells, while T cells were frozen until macrophage differentiation was complete. Monocyte-derived suppressive M2 macrophages were pre-treated with the 19L04c prior to the addition of allogeneic T cells, and the antibody was washed out prior to T-cell addition. T cells were not exposed to PSGL-1 antibody. Compared to the isotype control, cultures with 19L04c-treated macrophages produced higher levels of pro-inflammatory macrophages-derived cytokines including GM-CSF, TNFa, and IL-12 (Figure 2C(a–c)) and T-cell-derived IFNg (Figure 2C(d)), as well as a higher proportion of proliferating T cells defined by Ki-67+ expression (Figure 2C(e)) and activated T cells defined CD25 positivity (Figure 2C(f)). Since the T cells were not treated with anti-PSGL-1, the data suggest that T-cell activation is caused by the 19L04c-induced repolarization of the suppressive macrophages. Both SEB-PBMC and MRL data demonstrate that the effect of anti-PSGL-1 on macrophage function translates to coordinated immune responses, consistent with the hypothesis that PSGL-1 blockade on TAMs may lead to a coordinated anti-tumor microenvironment.

### 3.3. 19L04c Induces Strong Anti-Tumor Activity in a Model with an Aggressive Tumor Implanted in Humanized Mice

**Figure 3:**
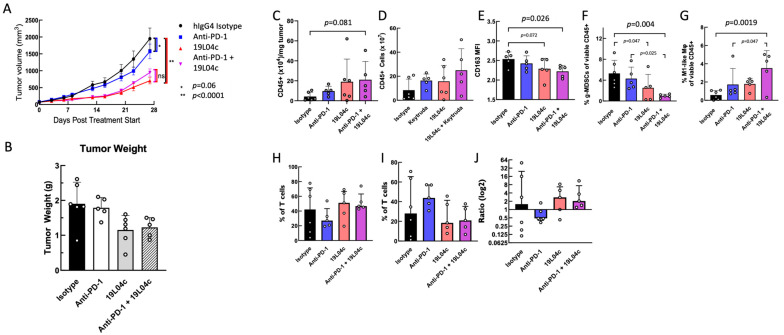
19L04c induces a pro-inflammatory microenvironment and leads to the suppression of tumor growth in a humanized mouse model. Tumor volume (**A**) and tumor weights in grams (**B**) of melanoma tumors in NGS-SGM3-BLT mice treated with 19L04c, Pembrolizumab, 19L04c plus Pembrolizumab combination or an Isotype control. Data are presented as the mean tumor volume ± standard deviation of the mean for all dose groups. Isotype n = 6, 19L04c n = 6, Pembrolizumab n = 5, 19L04c + Pembrolizumab n = 5. Dosing was initiated on Day 0. “ns” indicates no significance. (**C**) Total leukocytes per milligram of tumor calculated as the number of CD45 leukocytes divided by the weight of the tumor from each mouse. (**D**) The absolute number of leukocytes per tumor is calculated as the percent of viable CD45-positive leukocytes measured by flow cytometry multiplied by the total dissociated cell values in cells/mL. (**E**) MFI of the M2 marker CD163 on the total population of CD45^+^CD11b^+^CD14^+^CD3^−^ macrophages identified using UMAP dimensionality reduction. The percentage of (**F**) granulocytic MDSCs identified as CD33^+^ CD11blow MHCII low SSC high, (**G**) M1-like macrophages identified as CD33^+^ CD86^+^ MCHII^+^ CD163^−^, (**H**) effector T cells identified as CD8^+^ CD3^+^, (**I**) regulatory T cells identified as CD8^−^CD25^+^CD3^+^ by flow cytometry. (**J**) The ratio of effector to regulatory T cells based on the percentage of each subset.

To assess the effect of anti-PSGL-1 in vivo on tumor growth, 19L04c was evaluated in a humanized mouse study. We needed to use mice with the human immune system, as our antibody does not react to mouse PSGL-1. Data with a surrogate anti-mouse PSGL-1 antibody have been recently published [21]. We selected a tumor model based on the high content of TAMs of human origin naturally attracted by this tumor (personal communication by Dr. Brehm and [21,31]). This model is quite resistant to T-cell checkpoint inhibition. Treatment of the PDX-melanoma humanized NSG-SGM3-BLT mice with 19L04c led to statistically significant inhibition of tumor growth both as a monotherapy treatment and in combination with Pembrolizumab (Figure 3A,B and Figure S4) compared to the control group treated with an isotype-matched irrelevant antibody. 19L04c monotherapy and 19L04c combination treatment with Pembrolizumab were indistinguishable in this model (*p* = 0.5656) at the level of tumor growth inhibition. Pembrolizumab monotherapy worked as expected in this model leading to tumor growth inhibition that did not meet statistical significance (*p* = 0.0604, Figure 3A). In addition to seeing a large effect on tumor growth inhibition in the model, it was also gratifying to see that 19L04c did not lead to any adverse reactions when used alone or in combination with Pembrolizumab.

To analyze the effect of the antibody treatment on immune cell populations in the tumor, we performed a flow cytometric analysis of the tumor and spleen at the end of the study. The cells were stained to enumerate multiple lymphocyte and leukocyte populations as well as to characterize their activation status to broadly survey changes mediated by different therapies. The data were analyzed both by classical flow cytometry analysis as well as by multiparametric unbiased by the user analysis using UMAP clustering. Conclusions derived by both methods of analysis were in good agreement. Interestingly, many atypical changes were revealed by multiparametric analysis due to the absence of biasing the analysis by pre-determined gating strategies. We have observed that tumors from mice treated with 19L04c as a monotherapy or in combination with Pembrolizumab had a higher number of CD45^+^ leukocytes per gram of tissue (Figure 3C) translating into a higher absolute amount of CD45^+^ cells (Figure 3D) per tumor even though the tumors were smaller (Figure 3B), indicative of immune-mediated tumor destruction. Furthermore, similar to the observed shift in profile from an M2-like to an M1-like macrophage induced by 19L04c observed in multiple in vitro assays, 19L04c treatment led to a significant decrease in an M2-like phenotype of the tumor-infiltrating CD11b^+^CD14^+^ macrophage population as indicated by a decrease in expression levels of CD163, a marker of M2-like macrophages (Figure 3E), and in a decrease in suppressive granulocytic macrophages identified by CD33^+^CD11b^low^MHCII^low^SSC^high^ (Figure 3F). In addition, there was an increase in M1-like macrophages as identified by CD33^+^CD86^+^MHCII^+^CD163^−^ by 19L04, which reached significance in combination with Pembrolizumab treatment (Figure 3G).

T-cell populations were also modulated, likely because of macrophage repolarization. There was an increase in total CD45^+^CD3^+^CD8^+^ T (Teff) cells (Figure 3H) and a decrease in Treg (CD45^+^CD3^+^CD25^+^CD8^−^) cells (Figure 3I) resulting in an increase in the ratio of Teff to Treg cells (Figure 3J). Data from the humanized mouse model presented here support the hypothesis that VTX-0811 functionally switches macrophages in tumors from an M2-like to a more M1-like phenotype and activates T cells in the tumor, both of which contribute to a reduction in tumor growth.

### 3.4. 19L04c Induced Anti-Tumor Inflammatory Response in Ex Vivo Patient-Derived Human Tumors

To determine if VTX-0811 could re-polarize a primary human tumor microenvironment to an anti-tumor state, 19L04c was tested in primary human tumor cultures. These tumor cultures come straight from the surgery suite and contain all cell populations present in the tumor microenvironment, such as tumor, immune, and stromal cells, and other factors present in a patient tumor’s microenvironment, such as tumor-specific mixtures of cytokines, chemokines, and growth factors, as well as multiple known and unknown ligands for the relevant immune-suppressive targets. Since we and others always have seen PSGL-1 expression on TAMs [32], and TAMs are present in the vast majority of patient tumors, tumor tissues from all solid tumor types were accepted. Sixteen tumors were treated, with each tumor treated with an isotype control antibody, Pembrolizumab, and with 19L04c. After eliminating five tumors from the analysis based on the non-viability of the tissue across all treatment groups, the resultant data set consists of tumors from 11 patients representing 5 different cancer types that sampled the continuum of what is considered immunogenic and non-immunogenic tumor types. It has been established that a response of at least 30% induction of a single key cytokine (e.g., IFNg) in the ex vivo tumor model is correlative with clinical responses [33,34]. 19L04c demonstrated a consistent upregulation of multiple cytokines and chemokines across multiple tumors in a similar range, demonstrating a potent anti-PSGL-1-mediated tumor response across multiple tumors and tumor types. Induction of inflammatory responses in the tumor cultures by 19L04c was assessed as percent induction (% of isotype control) of three signatures of pro-inflammatory cytokines and chemokines. These three signatures consist of myeloid-focused cytokines (TNFa, GM-CSF, and IL-1b), chemokines (CCL3, CCL4, CCL5, CXCL9, and CXCL10), and an effector T-cell-derived cytokine (IFNg) and represent important aspects of a macrophage-based anti-tumor immune response.

**Figure 4:**
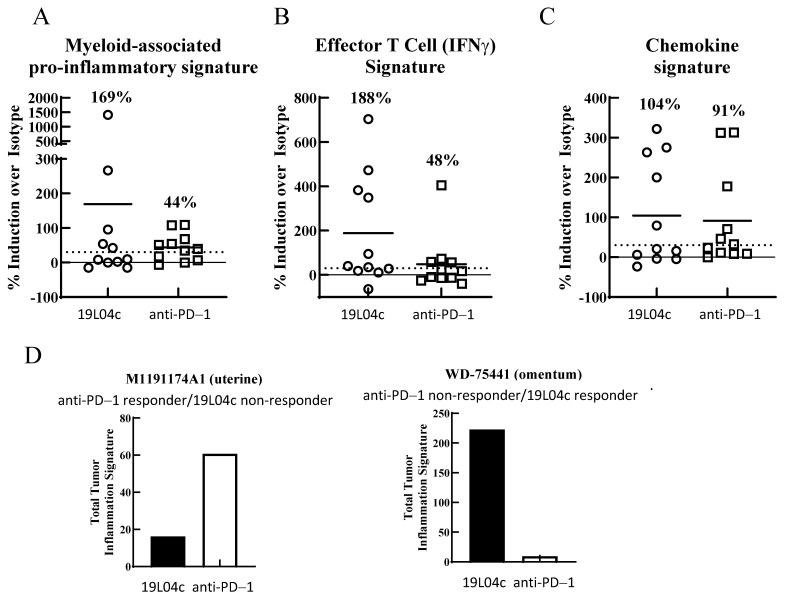
19L04c induces a pro-inflammatory signature in ex vivo human tumor cultures. Percent induction of a (**A**) myeloid-associated pro-inflammatory cytokine signature (TNFα, IL-1β, GM-CSF), (**B**) an effector T-cell signature (IFNγ), and (**C**) a chemokine signature (CCL3, CCL4, CCL5, CXCL9, CXCL10) in human tumors treated with 19L04c or Pembrolizumab compared to an isotype control (n = 11 tumors). Solid horizontal lines (and numbers above the data sets) indicate the mean values in each treatment group. The dotted lines mark 30% induction levels. (**D**) Total tumor inflammation signature (IFN-γ, TNFa, IL1b, GM-CSF, CCL3, CCL4, CCL5, CXCL9, CXCL10) in two individual tumors: M1191174A1 (uterine), a 19L04c non-responder with response to Pembrolizumab (**left**) and WD-75441 (omentum), a Pembrolizumab non-responder with response to 19L04c (**right**).

First, the 19L04c-induced secretion of the myeloid-focused cytokines is expected when the antibody induces repolarization of TAMs, resulting in the production of pro-inflammatory cytokines. 19L04c induced a greater than 30% increase in these cytokines in 5 out of 11 tumors while Pembrolizumab showed an effect in 7 out of 11 tumors. However, the average effect of 19L04c was stronger than that of Pembrolizumab (169% vs. 44%; Figure 4A).

Second, induction of the key T-cell-derived cytokine, IFNg, suggests that repolarization of TAMs translated to the activation of T cells within the tumor culture. 19L04c treatment induced an increase in IFNg by T cells across 7 out of 11 tumors compared to Pembrolizumab, which has a different mechanism of action that leads directly to greater IFNg production by T cells. Pembrolizumab produced an effect in 4 out of 11 tumors. Also, the effect seen from 19L04c is on average stronger than that from Pembrolizumab treatment (188% vs. 48%) (Figure 4B).

Third, chemoattraction signature was also modeled by combining an effect across several chemokines that are endogenously expressed in “hot” T-cell-infiltrated tumors and considered to be indicative of response to T-cell checkpoint inhibitors: CCL3, CCL4, CCL5, CXCL9, and CXCL10 [35,36,37]. 19L04c induced secretion of the chemokines within the chemokine signature in 5 out of 11 tumors while Pembrolizumab showed an effect in 6 out of 11 tumors. However, the effects of 19L04c and Pembrolizumab on average across all tumors were similar (104% vs. 91%; Figure 4C). The induction of a broad panel of chemoattractive chemokines by 19L04c suggests that, in a while organism setting, 19L04c would facilitate the recruitment of naïve immune cells to the tumor, which would lead to perpetuation of the anti-tumor immune response initiated by TAM repolarization.

To directly compare 19L04c and Pembrolizumab across all three mechanisms, the three immune signatures were combined in a total tumor inflammatory signature consisting of all nine cytokines and chemokines: IFNg, TNFa, IL1b, GM-CSF, CCL3, CCL4, CCL5, CXCL9, and CXCL10. Using this analysis, Pembrolizumab had a response in two tumors (uterine (Figure 4D) and kidney (Figure 4)) that did not show a response to 19L04c. The reverse outcome also occurred in two tumors (omentum (Figure 4D) and a different kidney tumor (Figure 4)). Tumors that did not respond to Pembrolizumab had greater responses to 19L04c compared to those who did respond to Pembrolizumab. Figure 4D shows two examples of individual tumors. The panel on the left belongs to the tumor with the strongest response to Pembrolizumab which did not show a response to 19L04c (uterine). The panel on the right shows the strongest response to 19L04c in a tumor with no response to Pembrolizumab (omentum). Overall, the ex vivo tumor culture data demonstrate that blockade of PSGL-1 offers the potential to provide clinical benefits to cancer patients in monotherapy due to reprogramming the macrophage infiltrate residing in the tumor bed and thus overcoming the local immunosuppressive milieu. Biomarker questions related to different mechanisms, such as T-cell activation through PD-1 inhibition versus macrophage repolarization through PSGL-1 inhibition, will need larger experiments to sample cancer diversity and may ideally be addressed in the clinic.

### 3.5. 19L04c Molecular Pathway Analysis

**Figure 5:**
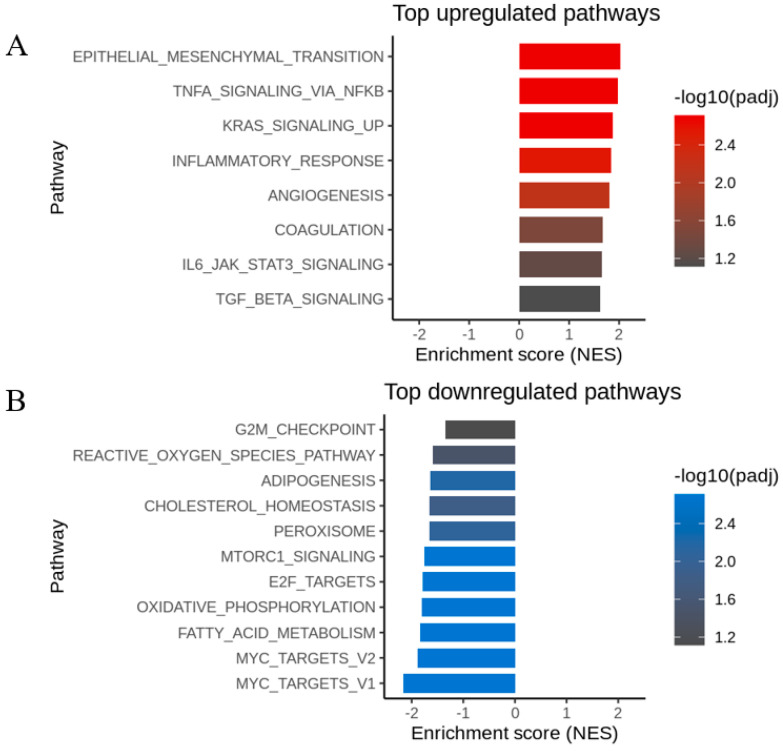
19L04 treatment leads to inflammatory shifts in primary M2c macrophages. M2c macrophages obtained from PBMCs of four donors treated with 19L04c or isotype control were subjected to RNAseq profiling. Differential gene expression was completed between the two treatment groups paired by the donor. Gene Set Enrichment Analysis was used to analyze the gene expression differences between the treatment groups. (**A**) 19L04c treatment mostly upregulated genes enriched in general inflammatory responses and TNFα signaling. (**B**) 19L04 treatment led to the downregulation of metabolic pathways, such as fatty acid metabolism and oxidative phosphorylation, and Myc targets pathways, all known to be associated with suppressive macrophages.

To broaden the scope of the pro-inflammatory response beyond secreted mediator analysis and further explore the MOA of anti-PSGL-1, M2c macrophages obtained from PBMCs of four donors treated with 19L04c or an isotype control were subjected to RNAseq analysis. In this highly suppressive culture, and consistent with the secreted mediator data, 19L04c upregulated genes enriched in general inflammatory responses and TNFa signaling (Figure 5A). We also observed downregulation of metabolic pathways, such as fatty acid metabolism and oxidative phosphorylation, and Myc targets pathways, all known to be associated with suppressive macrophages (Figure 5B) [6,38,39,40,41,42,43]. The secreted mediator and pathway analysis supports that 19L04c leads to a shift from suppressive M2 macrophages towards an M1 anti-tumorigenic macrophage phenotype.

### 3.6. VTX-0811 Effects Are Limited to Sites with Ongoing Immune Response; It Does Not Induce Inflammation in Naïve Unstimulated PBMCs, T Cells, or Neutrophils

**Figure 6:**
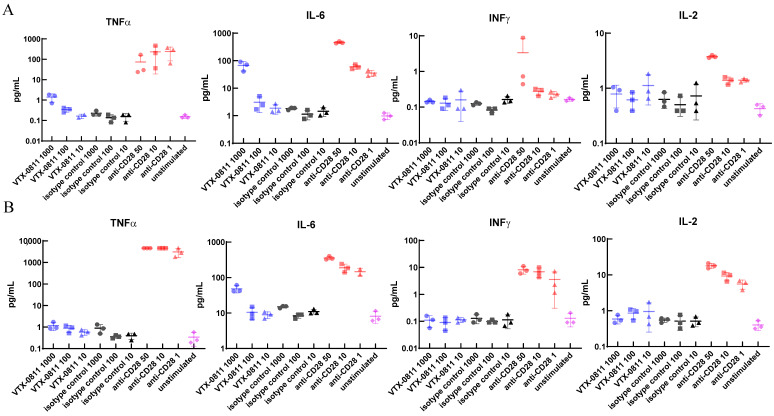
VTX-0811 does not elicit cytokine release from naïve unstimulated PBMCs. Secreted cytokine levels (pg/mL) in response to treatment of unstimulated human PBMC with soluble (**A**) or plate-bound (**B**) 19L04c, anti-CD28, or an isotype control measured by Luminex. For the plate-bound method, tissue culture treated, flat bottom, 96-well plates were coated with 100 µL/well of F(ab′)_2_ goat anti-human IgG, IgM (H + L) (10 µg/mL) at 4 °C for 24 h, washed, and then treated with VTX-0811, IgG4 isotype control, or anti-CD28 for 24 h prior to adding the cells. 19L04h and isotype control were evaluated at 10, 100, and 1000 µg/mL; anti-CD28 positive control was evaluated at 1, 10, and 50 µg/mL. Data shown are mean values ± standard deviation from six donors.

One concern when working with a first-in-class antibody is that it may have the capacity to induce an unintended pro-inflammatory response; therefore, the antibody should be tested to determine whether it can activate naïve human immune cells in the blood. A clean toxicity profile in cynomolgus macaques with near indistinguishable affinity of the antibody to human and cynomolgus PSGL-1 is reassuring (see data below) but testing on primary human cells is paramount. We tested if VTX-0811 is inducing activation of unstimulated-resting human PBMCs in vitro using TGN1412 anti-CD28 antibody as a positive control. VTX-0811 did not produce appreciable increases in any cytokine evaluated in either the soluble (Figure 6A) or plate-bound assay (Figure 6B) format, even though multiple cell types express PSGL-1 in human peripheral blood [21,44].

Anti-CD28 antibody, as expected, led to the induction of multiple cytokines in both the soluble (Figure 6A) and plate-bound (Figure 6B) formats. However, in this assay, a dose–response of the anti-CD28 antibody was not apparent for all cytokines perhaps indicating that 1 µg/mL anti-CD28 may be close to producing maximal cytokine response in human unstimulated PBMCs. In contrast, anti-PSGL-1 antibodies, 19L04c and VTX-0811 at a 1000-fold higher concentration (1000 µg/mL), produced no appreciable increase in any cytokine evaluated. This concentration of VTX-0811 was chosen to be approximately equivalent to the projected maximum serum concentration expected from intravenous dosing at the highest proposed dose in the Phase 1a dose escalation arm of a future clinical study. The lack of any substantial cytokine release from PBMCs at a concentration of 1 mg/mL indicates no appreciable agonist activity for VTX-0811in an in vitro assay with unstimulated PBMCs under conditions where agonistic activity is seen for an anti-CD28-positive control antibody.

**Figure 7:**
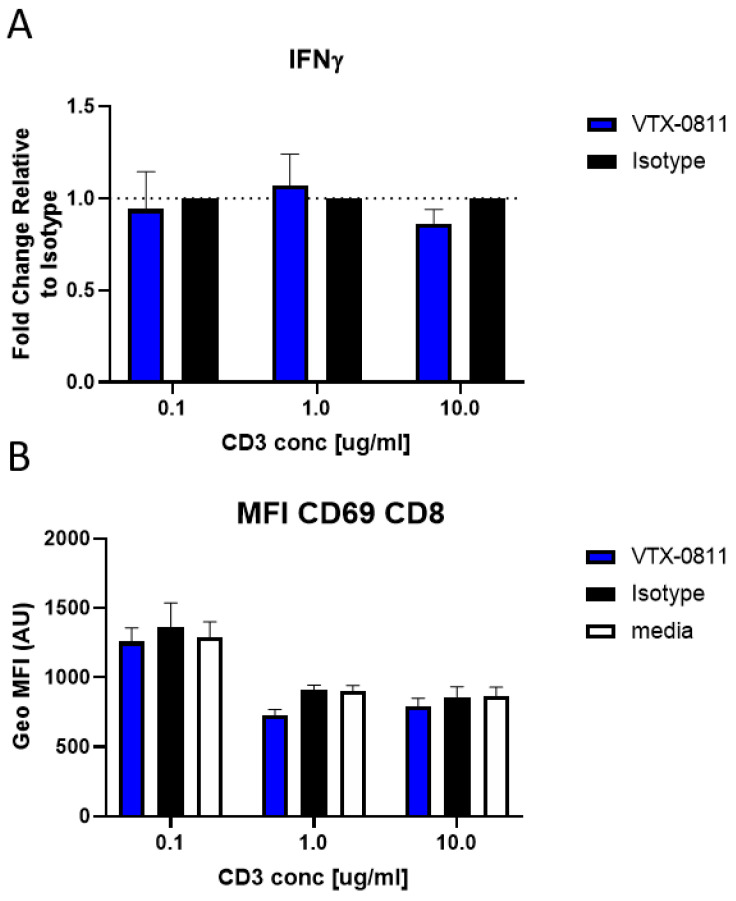
VTX-0811 has minimal direct impact on T-cell function. Purified human T cells, activated with CD3 and CD28, were co-incubated with VTX-0811 antibody. Changes in IFNγ in the supernatant of the cultures (**A**) as well as changes in the MFI of CD69 staining intensity on CD8+ T cells (**B**) were compared to control-treated cells. Data are shown as mean values ± standard deviation from six donors.

We also wanted to test if VTX-0811 has the potential to influence activated T cells directly when no other cells are present. To address this question, we tested if VTX-0811 has any impact on primary T cells activated with anti-CD3/CD28 antibodies. No effect of VTX-0811 was observed on T cells only containing cultures pre-activated with anti-CD3/CD28 (Figure 7).

**Figure 8:**
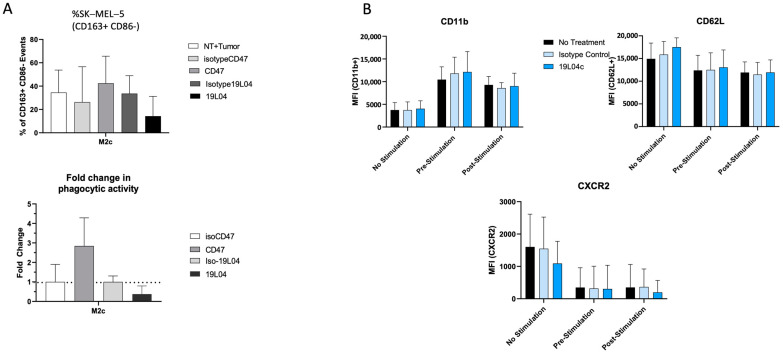
VTX-0811 has a modest effect on phagocytic activity and does not have an effect on human neutrophil activation. (**A**) M2c macrophages were differentiated and polarized from human blood monocytes. After polarization, the M2c macrophages were pre-incubated for 30 min with 19L04c, anti-CD47, or their respective isotype controls and then cultured with pHrodo-stained SK-MEL-5 cells for 2 h. The fold change was calculated based on the percentage of pHrodo+ gated from CD163+CD86- macrophages compared to the isotype control for 19L04 or the isotype control for anti-CD47 for each donor. The data represent an average of values obtained across 3 donors with 2 technical replicates per donor. (**B**) Whole blood was incubated with Isotype control or 19L04c or left untreated. One set of samples remained unstimulated (No stimulation). A second set was pre-activation for 15 min with fMLP prior to the addition of the antibody for 1 h (Pre-stimulation), and the third set was incubated with the antibody for 1 h prior to activation for 15 min with fMLP (Post-stimulation). The MFI of the indicated markers was assessed by flow cytometry and is representative of 3 donors.

Given that VTX-0811 is activating macrophages, it is important to address whether the antibody has an impact on the phagocytic ability of macrophages. To this end, we have tested whether M2c macrophages change their ability to phagocytose under the influence of VTX-0811. VTX-0811 decreases the ability of inhibitory macrophages to phagocytose, and the change is relatively small (Figure 8A).

Additionally, we wanted to see if naïve or preactivated neutrophils that express PSGL-1 are functionally changed by VTX-0811. To test it, we have treated naïve unstimulated, pre-stimulated or first exposed to VTX-0811 or the control antibody, and then stimulated cells. The levels of CD11b, CD62L, and CXCR2 were monitored on neutrophils, with the expected increase in CD11b, and decrease in CD62L and CXCR2 in case of activation (Figure 8B). VTX-0811 did not affect neutrophils under any of the conditions tested (Figure 8B). The data described in this section demonstrate that VTX-0811 does not activate naïve PBMCs, does not activate naïve or stimulated T cells under the conditions tested, and does not influence naïve or activated neutrophils, likely relying on the activated macrophages to initiate its broad repolarizing effect.

### 3.7. VTX-0811 Demonstrates Safety in NHPs Study up to the Highest Doses Tested

After demonstrating the efficacy of VTX-0811 in preclinical models, we turned to assessing its drug-like properties. Stability, solubility, and ability to saturate the receptor with doses that can be easily administered to patients and the safety of the drug are paramount to enable further development. Given that VTX-0811 is an antibody on a natural human IgG4/kappa backbone, a lot of the characteristics of the antibody do not surprisingly resemble the classical human IgG4 profile.

**Figure 9:**
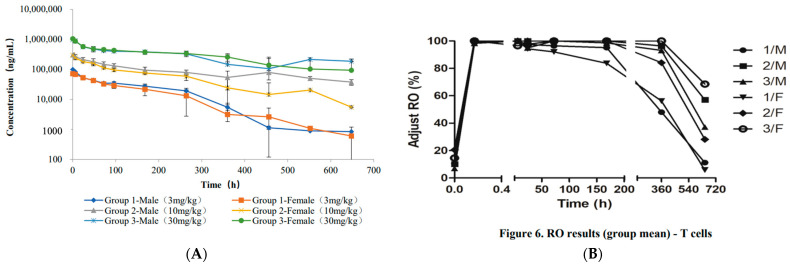
PK and RO of VTX-0811-CHO in cynomolgus monkeys. (**A**) PK of VTX-0811 in NHP. In the single-dose non-GLP compliant PK study, 3 male and 3 female cynomolgus monkeys (n = 1/group) received a single IV dose of VTX-0811 at 3, 10, or 30 mg/kg administered as a 30 min infusion. The concentration of the drug in the blood is plotted. Cmax was observed at 0.5 h post dose initiation for all doses. Cmax increased in a dose-proportional manner. (**B**) Target engagement for PSGL-1 was determined using a receptor occupancy method. VTX-0811 presented essentially dose-dependent binding to PSGL-1 on the surface of both T-cell (CD3^+^) and leukocyte (CD11b^+^) in whole blood samples collected in the single-dose PK study. For both cell types, PSGL-1 RO was near the peak or peaked at 0.5 h post infusion. RO was high at 79.24–100% and remained high at 0.5–168 h post-dosing for all dose levels assessed in both cell populations. For 3, 10, and 30 mg/kg doses, receptor occupancy returned to pre-dose levels at 648 h. Receptor occupancy data correlated with serum concentration, both declining at roughly 168 h, though the decline in RO was slower than the decline in exposure.

Before going into the safety toxicology study, the PK of VTX-0811 was evaluated in a single-dose study in cynomolgus monkeys. The PK data demonstrated that the T_1/2_ of VTX-0811 ranged from 42 (F) to 69 (M) h for the 3 mpk group, from 83 (M) to 110 (F) h for the 10 mpk group, and 35 h for the 30 mpk group. These data indicated that VTX-0811 showed a typical human antibody PK profile in an NHP study and had good levels throughout the study (Figure 9A). 

Furthermore, VTX-0811 showed high levels of receptor occupancy (RO), especially through 360 h. When VTX-0811 injection was administered at a dose of 3 mg/kg, RO on T cells in peripheral blood remained at a high level from 10 min to 168 h after dosing, with decreased RO in some animals at 360 h, and RO recovered to pre-dose levels in all animals at 648 h. At the doses of 10 and 30 mg/kg, RO on T cells in peripheral blood remained at a high level from 10 min to 360 h after dosing, and while RO levels decreased to pre-dose levels in some animals at 648 h (2 out of 6 animals in Group 2, and 2 out of 6 animals in Group 3), RO remained high in the remainder of animals in both dose groups (Figure 9B). These data demonstrated that PSGL-1 can be saturated by VTX-0811 at doses at or above 1 mg/kg and decreases in a dose-dependent manner. These data were very instructive for clinical study design, where we do not expect the need to dose higher than 10–15 mg/kg every 2–3 weeks, as such doses are expected to provide continuous near complete receptor occupancy.

### 3.8. No Test Article-Related Observations Were Recorded for VTX-0811

For toxicology assessment, VTX-0811 was dosed at 25, 75, and 200 mg/kg weekly for 4 weeks totaling 5 doses administered on Day 1, Day 8, Day 15, Day 22, and Day 29. Each dose cohort consisted of 5 male and 5 female animals. Doses were chosen after a small dose range finding study with dosing up to 300 mg/kg indicated no adverse events. For all VTX-0811 dosed and the placebo groups, a subgroup of n = 3 female and n = 3 male monkeys was sacrificed immediately after dosing completion (at Day 30), and a subgroup of n = 2 female and n = 2 male monkeys after a period of recovery (Day 58).

No test article-related changes were noted in clinical observations, draize evaluation, body weight data, food consumption, ophthalmology examination, blood pressure, ECG parameters, respiratory function parameters, clinical pathology parameters (clinical chemistry, hematology, coagulation, or urinalysis), complement (C3 and C4) levels, IgA and IgM levels, gross observations, absolute and relative organ weight parameters, or histopathology evaluations. VTX-0811 appeared to be exceedingly well tolerated, and the dose with no adverse events was assigned as the highest dose administered, which is likely an underestimate for the dose.

At the level of soluble mediators, serum analysis showed that VTX-0811 injection did not induce the production of IL-2, IL-4, IL-5, TNF-α, IL-6, and IFN-γ in the peripheral blood of the cynomolgus monkeys at any dose at the time points tested. At the cellular level, there were no significant VTX-0811-mediated differences in the number of total, cytotoxic, DP, DN, or helper T cells; B or NK cells did not show any differences either. In conclusion, the no-observed-adverse-effect-level (NOAEL) and highest non-severely toxic dose (HNSTD) is 200 mg/kg/dose, equivalent to the highest tested dose.

## 4. Discussion

A clinically diagnosed tumor has overcome multiple resistance mechanisms with the biggest one of avoiding immune response to itself under conditions of smoldering tissue damage, lack of nutrients, lack of blood flow, modified pH, and continuous attempts of the immune system to eliminate the transformed tissue. The role of macrophages in the tumor microenvironment should not be underestimated: These cells need to be controlled by the tumor to avoid the initiation of an active pro-inflammatory anti-tumor response, to prevent the initiation of active pro-inflammatory chemotaxis, the tumors need to engage macrophages in tumor niche formation, organize neo-angiogenesis. Macrophages are located deep in the tumor bed or at the perimeter of the tumor, are modulated by the tumor, and are actively involved in phagocytosing tumor cell debris, thereby presenting tumor-associated antigens but are influenced by the tumor to become suppressive macrophages. Several different approaches have been suggested to influence TAMs: depletion, activation of the do-not-eat-me signal, and repolarization [9]. As evident from CSF-1R inhibition, depletion seems to activate CSF-1 production and quick repopulation [45]. In addition, the depletion approach robs the tumor of many antigen-presenting cells instrumental in initiating a productive anti-tumor immune response. Activation of the do-not-eat-me signal has led to clinical success when used in combination with antibodies targeting a tumor antigen such as HER2, CD33, or CD20, demonstrating that an additional push towards a pro-inflammatory response delivered by the tumor-targeting antibody is needed in addition to stimulating phagocytic properties [46,47]. Specific repolarization approaches are only now entering mid-stage clinical development, with very exciting clinical data from the MK-3840 clinical trial [25], demonstrating responses in patients who have failed all available therapies, including PD-1/PD-L1 blockade. Clinical responses in solid cancer patients have also been shown for the Clevel-1 targeting antibody Bexmarilimab that induces macrophage repolarization [48].

We have previously demonstrated that PSGL-1 is an inhibitory checkpoint expressed on suppressive human TAMs and M2 macrophages, which promote a pro-tumorigenic state [21]. The critical role of PSGL-1 in maintaining the macrophages in a suppressed state was shown by the siRNA knockdown of PSGL-1 on human macrophages. Furthermore, attenuating PSGL-1 activity led to repolarization of the macrophages towards an M1 phenotype, an important quality of an anti-tumor microenvironment [21].

In this study, we identified an antibody that mimics the functional effect of siRNA knockdown of PSGL-1. Various immunization methods were utilized to make mouse hybridomas including a recombinant full-length PSGL protein, N-terminal peptide, or DNA encoding the full-length protein to yield a broad set of antibodies recognizing multiple epitopes on PSGL-1 molecule. These various methods yielded over 7500 hybridomas, from which 11 representing multiple epitope bins determined by antibody cross-blocking experiments by ForteBio Octet and ELISA, where antibodies that cross-compete for PSGL-1 binding are binned together. Representative antibodies from these 11 hybridomas were sub-cloned and subjected to secondary screening. Based on binding properties to the recombinant PSGL-1 and primary cells and the ability to induce GM-CSF from antibody-treated M2 cells, clone 19L04 was identified as a lead for further development.

19L04c is a chimeric antibody that consists of the murine 19L04 variable regions and human IgG4 (S228P) heavy and kappa light chain constant region sequences. 19L04c bound to recombinant human PSGL1-His EC50 of 0.449 mM. Functionally, 19L04c was shown to repolarize M2-suppressive macrophages towards a potent pro-inflammatory state characterized by secretion of TNFa, IL-1b, IL6, and GM-CSF. This effect on macrophages translated into complex immune response systems including the SEB-PBMC and MLR assays, where 19L04c induced the secretion of the same pro-inflammatory mediators secreted from 19L04c treated human M2c macrophages. Furthermore, in the complex immune cell assays, we also detected effector T-cell mediators such as IL-2 in the SEB-PBMC assay and IFNg in the MLR assay suggesting the repolarized macrophages led to the activation of effector T cells. However, although PSGL-1 is also expressed and has been shown to have direct functional effects on T-cell activation [14,15,17] and cell trafficking [12,13], neither of these attributes was impacted by PSGL-1 inhibition by our antibody (Figure 3 and Figure 7).

To assess if the observed effects of 19L04c in vitro on macrophages and complex immune responses translated in vivo, and if this modulation of an immune response affected tumor growth, 19L04c was evaluated in a humanized mouse model. Having shown in vitro that VTX-0811 and 19L04c have similar activity, 19L04c was used to evaluate the effect of blocking anti-PSGL-1 in vivo. 19L04c led to a significant decrease in tumor volume and a much deeper inhibition compared to Pembrolizumab. This decreased tumor volume correlated with an increase in total leukocytes and a re-direction of the tumor microenvironment to a pro-inflammatory anti-tumor state. 19L04c led to a decrease in M2-suppressive CD163 expressing macrophages and regulatory T cells and an increase in activated CD8 T effector cells in the tumors. Consistent with the in vitro data, the observed in vivo effects of 19L04c support the hypothesis that anti-PSGL-1 re-polarizes macrophages from an M2 towards an M1 functional profile and switches the tumor microenvironment to a pro-inflammatory phenotype leading to tumor growth inhibition. Similar increases in the effector to regulatory T-cell ratio were recently reported in tumors from syngeneic mouse models, where mice were treated with anti-PGSL-1 [22]. These effects were often even more pronounced in tumors from mice co-treated with anti-PD-1. Notably, effector T-cell mediators were elevated in the anti-PSGL-1 monotherapy and combination groups. It is important to mention that a different antibody to PSGL-1, as used in the published studies by other groups, might have a different mechanism of action and can lead to different activation mechanisms. Additionally, we have shown that our antibody does not activate T cells directly under the conditions tested and is not affecting naïve or pre-activated neutrophils that express PSGL-1.

From here we further assessed translational effects using human primary tumors. 19L04c was shown to induce a pro-inflammatory profile based on multiple mediator signatures encompassing pro-inflammatory cytokine and chemokines and T-cell activation. Combining all signatures into a final total tumor inflammatory signature score indicated that 19L04c led to an even higher degree of an anti-tumor response than that observed for Pembrolizumab. It should be noted that anti-PSGL-1-treated patient-derived tumor cultures demonstrated anti-tumor immune responses and T-cell activation even in tumors not responding to Pembrolizumab—a high unmet need patient population. As PSGL-1 is present in most patients across multiple indications, this antibody has a very broad potential applicability both as monotherapy and in combination with T-cell checkpoint inhibitors.

To progress 19L04 as the lead candidate, 19L04c was subsequently humanized (VTX-0811) via CDR-grafting into high-identity human germline frameworks and back-mutating to maintain structurally important mouse framework residues and to additionally remove select potential sequence liabilities. VTX-0811 binds to recombinant human bivalent PSGL-1 with a similar binding affinity as 19L04c and shows similar dose–response curves, and the EC50s demonstrate no reduction in binding following the humanization process.

VTX-0811 did not have any appreciable agonist activity on cytokine release from human primary unstimulated PBMCs, which addresses the FDA Guidance for “Immunogenicity Assessment for Therapeutic Protein Products” targeting cell surface receptors on cytokine release. This is a necessary requirement for developing antibodies aimed to enhance anti-tumor immune response.

## 5. Conclusions

In conclusion, we describe a first-in-class anti-PSGL-1 antibody with an excellent safety profile, VTX-0811. VTX-0811 inhibits PSGL-1 functionality on human macrophages, which leads to a switch of suppressive macrophages and the tumor microenvironment to a pro-inflammatory status. This, in turn, activates an anti-tumor state and suppression of tumor growth in a tough-to-treat preclinical model.

## Data Availability

Data are contained within the article or Supplementary Materials.

## Supplementary Materials

The following supporting information can be downloaded at https://www.mdpi.com/article/10.3390/cancers16162778/s1, Figure S1: PSGL-1 is upregulated by PD-1 blockade in melanoma patients; Figure S2: Anti-PSGL-1 mAbs are functional in both SEB and Macrophage Assays; Figure S3: VTX-0811 binds to recombinant and cell surface human PSGL-1, it also binds cynomolgus macaque PSGL-1; Figure S4: Individual spider graphs showing the effect of anti-PSGL-1 monotherapy or in combination with anti-PD-1 in a humanized mouse model.
