# Supplementary material for: Preclinical Efficacy of VTX-0811: A Humanized First-in-Class PSGL-1 mAb Targeting TAMs to Suppress Tumor Growth
Source: Cancers (Basel). 2024 Aug 6;16(16):2778. doi: 10.3390/cancers16162778 (PMC11352552; doi:10.3390/cancers16162778)
Supplement: Supplementary file 1 [file cancers-16-02778-s001.zip › cancers-3098371-supplementary.pptx]

## Slide 1
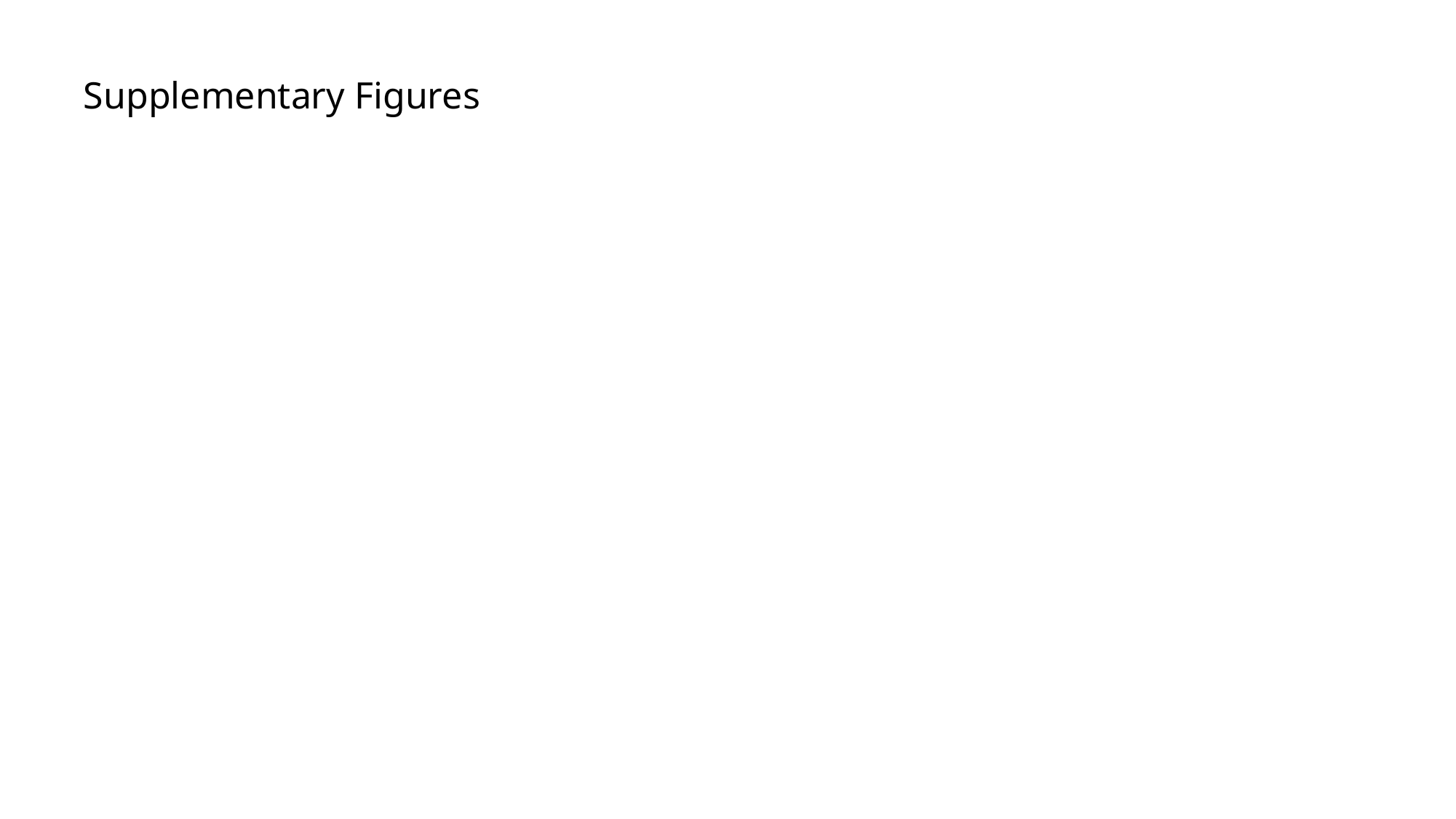

# Supplementary Figures
1

## Slide 2
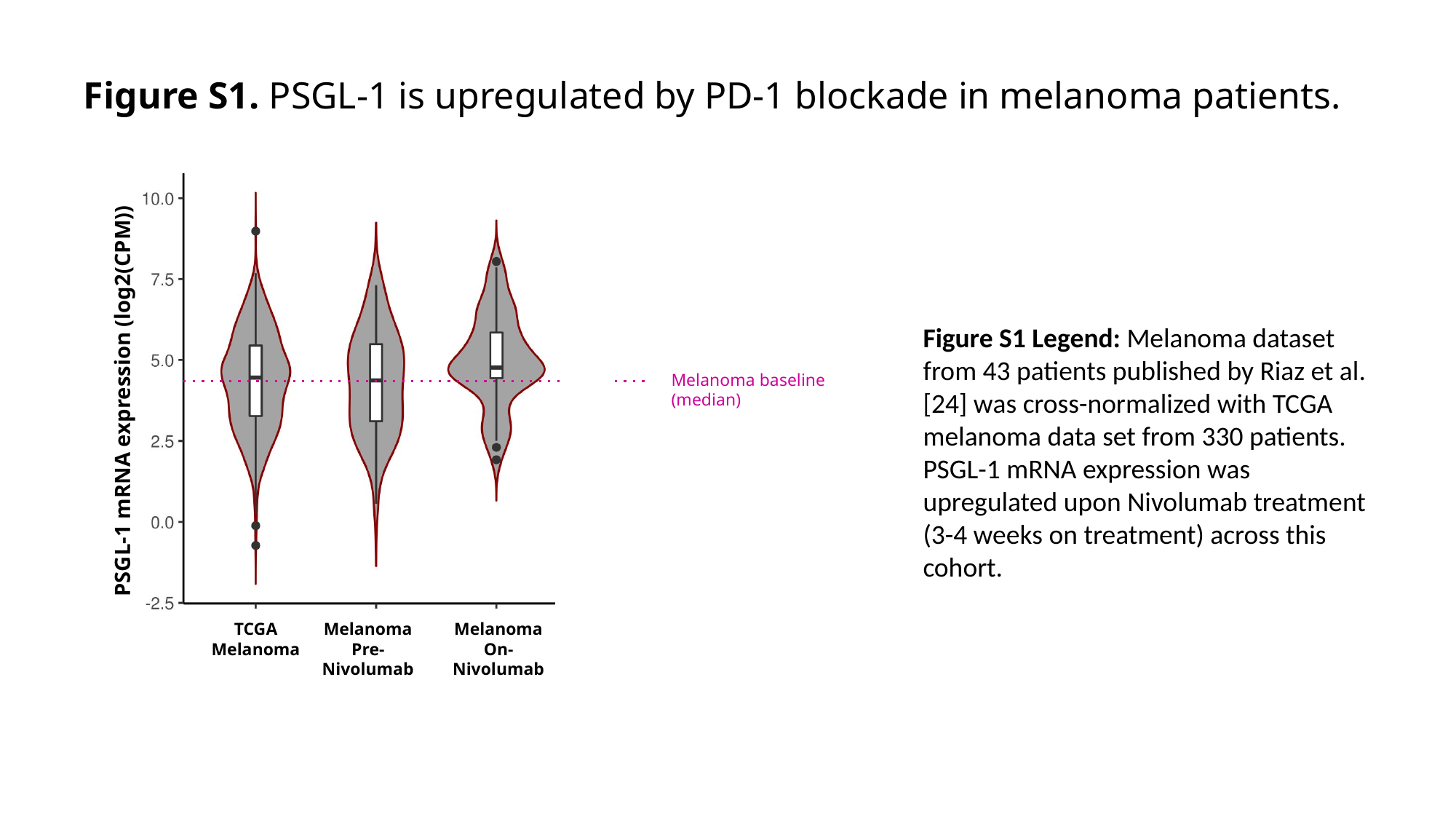

# Figure S1. PSGL-1 is upregulated by PD-1 blockade in melanoma patients.
Figure S1 Legend: Melanoma dataset from 43 patients published by Riaz et al. [24] was cross-normalized with TCGA melanoma data set from 330 patients. PSGL-1 mRNA expression was upregulated upon Nivolumab treatment (3-4 weeks on treatment) across this cohort.
Melanoma baseline (median)
PSGL-1 mRNA expression (log2(CPM))
TCGA
Melanoma
Melanoma
Pre-Nivolumab
Melanoma
On-Nivolumab
2

## Slide 3
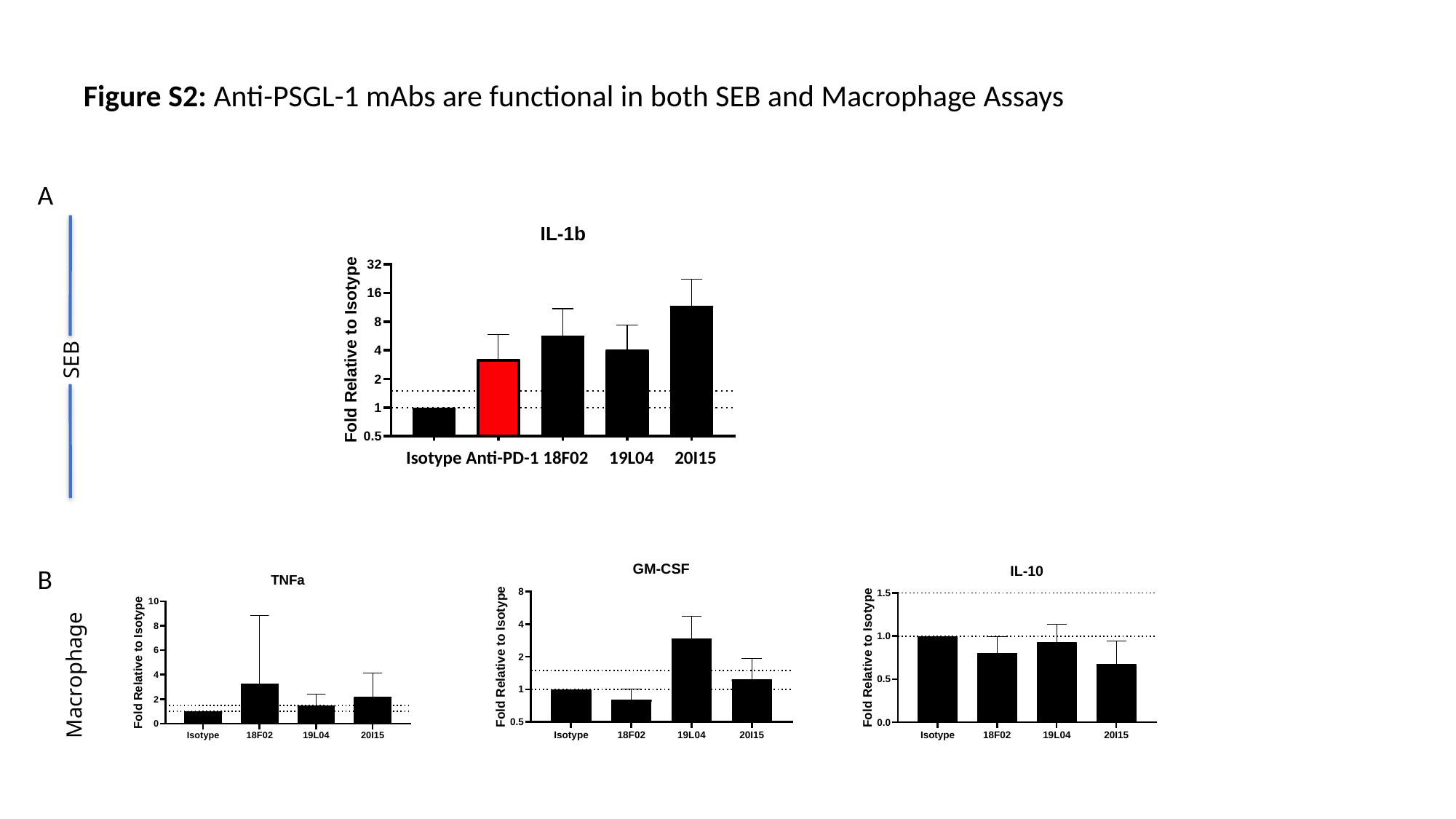

# Figure S2: Anti-PSGL-1 mAbs are functional in both SEB and Macrophage Assays
A
Isotype Anti-PD-1 18F02 19L04 20I15
SEB
B
Macrophage
Slide 3

## Slide 4
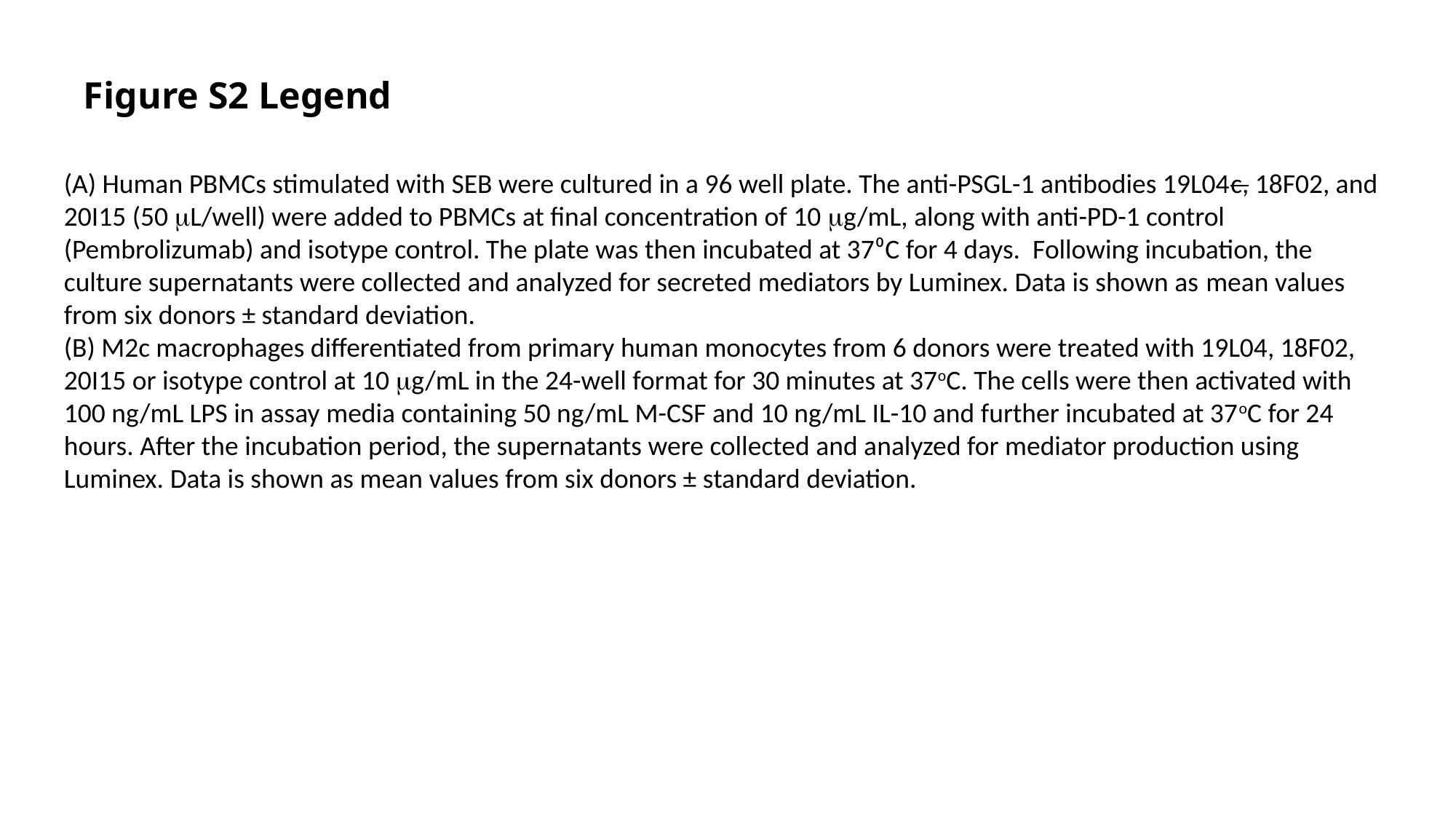

# Figure S2 Legend
(A) Human PBMCs stimulated with SEB were cultured in a 96 well plate. The anti-PSGL-1 antibodies 19L04c, 18F02, and 20I15 (50 mL/well) were added to PBMCs at final concentration of 10 mg/mL, along with anti-PD-1 control (Pembrolizumab) and isotype control. The plate was then incubated at 37⁰C for 4 days. Following incubation, the culture supernatants were collected and analyzed for secreted mediators by Luminex. Data is shown as mean values from six donors ± standard deviation.
(B) M2c macrophages differentiated from primary human monocytes from 6 donors were treated with 19L04, 18F02, 20I15 or isotype control at 10 mg/mL in the 24-well format for 30 minutes at 37oC. The cells were then activated with 100 ng/mL LPS in assay media containing 50 ng/mL M-CSF and 10 ng/mL IL-10 and further incubated at 37oC for 24 hours. After the incubation period, the supernatants were collected and analyzed for mediator production using Luminex. Data is shown as mean values from six donors ± standard deviation.
4

## Slide 5
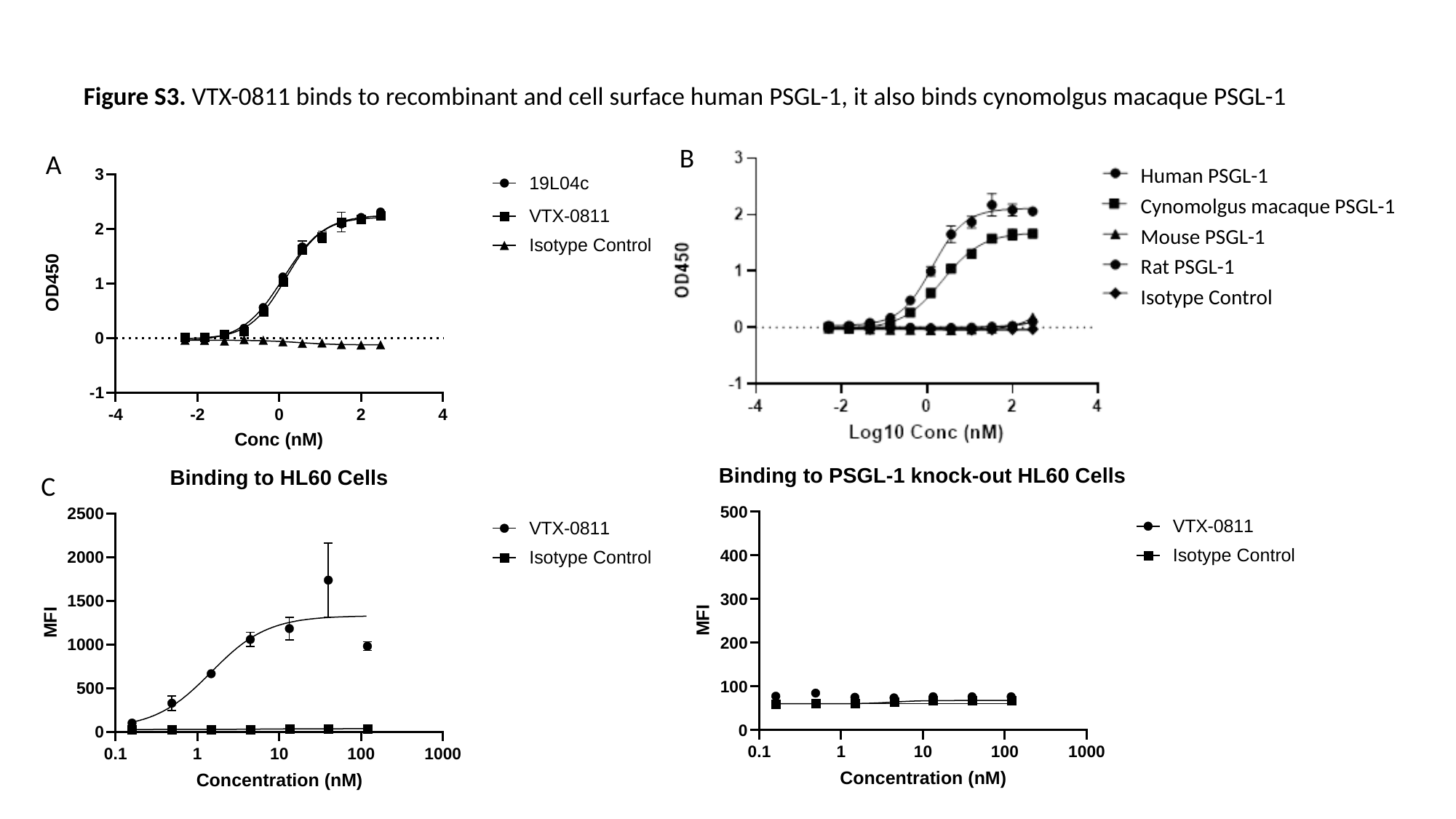

# Figure S3. VTX-0811 binds to recombinant and cell surface human PSGL-1, it also binds cynomolgus macaque PSGL-1
Human PSGL-1
Cynomolgus macaque PSGL-1
Mouse PSGL-1
Rat PSGL-1
Isotype Control
B
A
C
5

## Slide 6
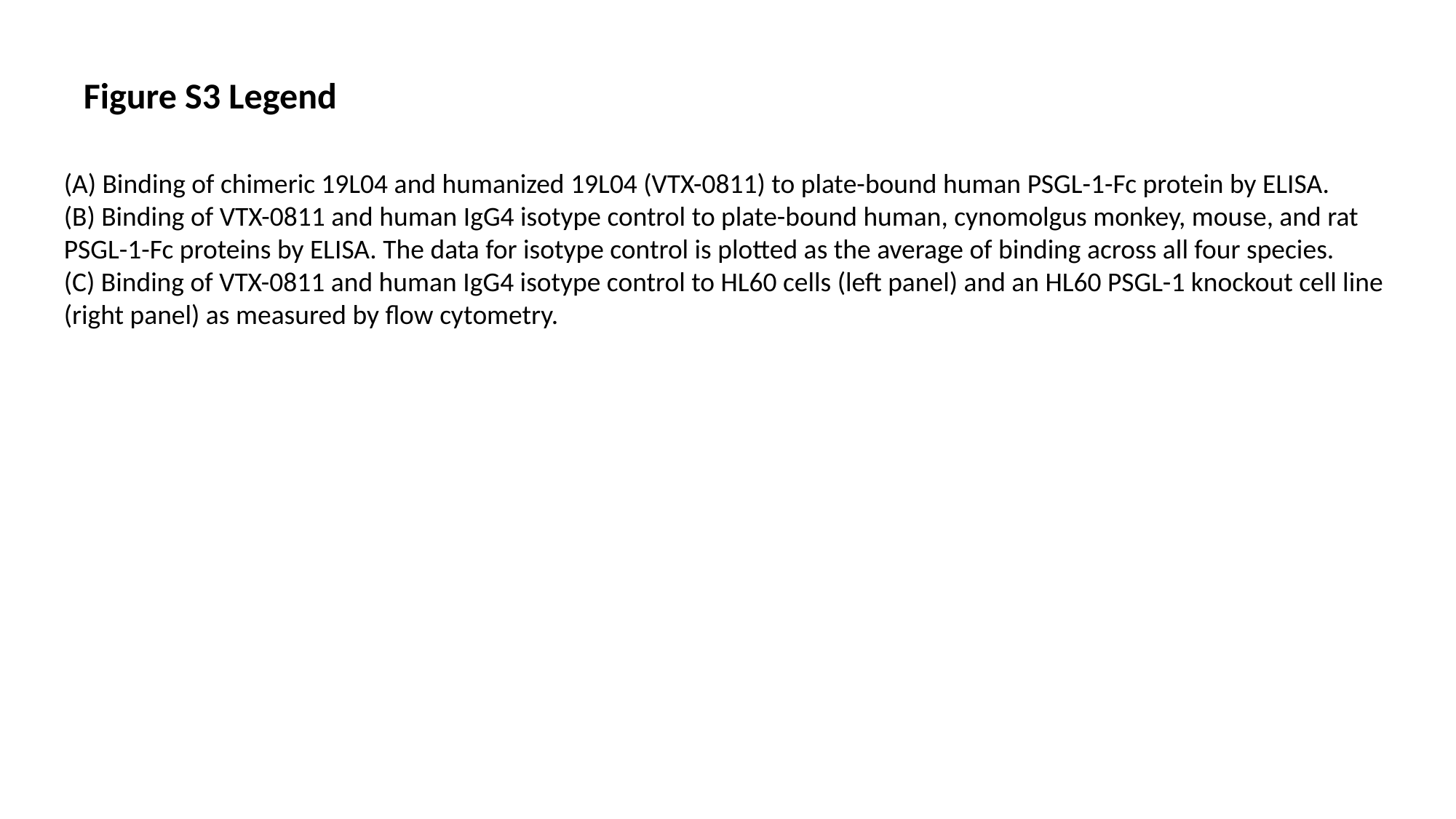

# Figure S3 Legend
(A) Binding of chimeric 19L04 and humanized 19L04 (VTX-0811) to plate-bound human PSGL-1-Fc protein by ELISA.
(B) Binding of VTX-0811 and human IgG4 isotype control to plate-bound human, cynomolgus monkey, mouse, and rat PSGL-1-Fc proteins by ELISA. The data for isotype control is plotted as the average of binding across all four species.
(C) Binding of VTX-0811 and human IgG4 isotype control to HL60 cells (left panel) and an HL60 PSGL-1 knockout cell line (right panel) as measured by flow cytometry.
6

## Slide 7
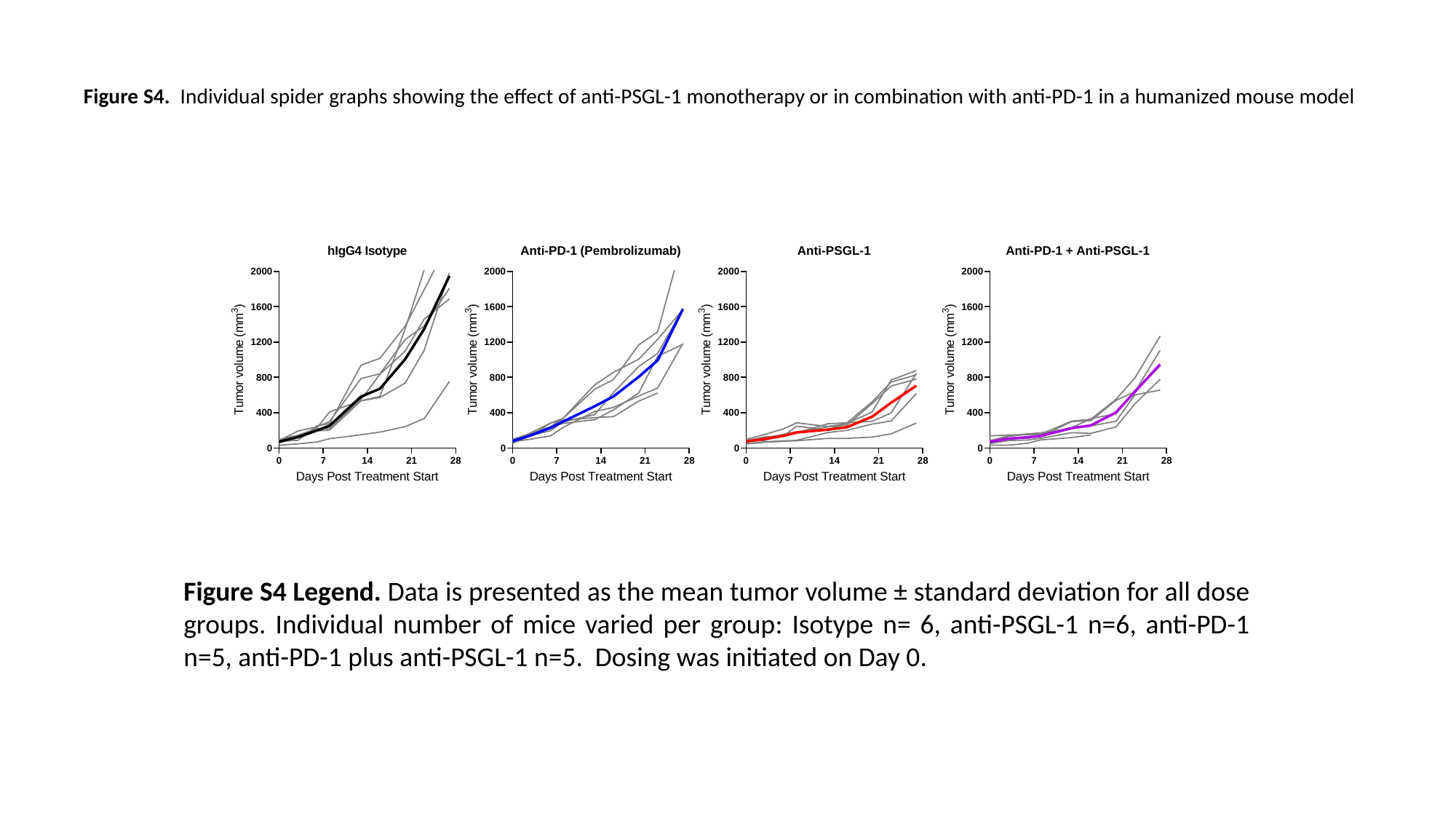

# Figure S4. Individual spider graphs showing the effect of anti-PSGL-1 monotherapy or in combination with anti-PD-1 in a humanized mouse model
Figure S4 Legend. Data is presented as the mean tumor volume ± standard deviation for all dose groups. Individual number of mice varied per group: Isotype n= 6, anti-PSGL-1 n=6, anti-PD-1 n=5, anti-PD-1 plus anti-PSGL-1 n=5. Dosing was initiated on Day 0.
7
